# In situ sensing physiological properties of biological tissues using wireless miniature soft robots

**DOI:** 10.1126/sciadv.adg3988

**Published:** 2023-06-07

**Authors:** Chunxiang Wang, Yingdan Wu, Xiaoguang Dong, Milena Armacki, Metin Sitti

**Affiliations:** 1 Physical Intelligence Department, Max Planck Institute for Intelligent Systems, Stuttgart 70569, Germany; 2 Institute for Biomedical Engineering, ETH Zürich, Zürich 8092, Switzerland; 3 Department of Mechanical Engineering, Vanderbilt University, Nashville, TN 37235, USA; 4 University Hospital Ulm, Ulm 89081, Germany; 5 School of Medicine and College of Engineering, Koç University, Istanbul 34450, Turkey

## Abstract

Implanted electronic sensors, compared with conventional medical imaging, allow monitoring of advanced physiological properties of soft biological tissues continuously, such as adhesion, pH, viscoelasticity, and biomarkers for disease diagnosis. However, they are typically invasive, requiring being deployed by surgery, and frequently cause inflammation. Here we propose a minimally invasive method of using wireless miniature soft robots to in situ sense the physiological properties of tissues. By controlling robot-tissue interaction using external magnetic fields, visualized by medical imaging, we can recover tissue properties precisely from the robot shape and magnetic fields. We demonstrate that the robot can traverse tissues with multimodal locomotion and sense the adhesion, pH, and viscoelasticity on porcine and mice gastrointestinal tissues ex vivo, tracked by x-ray or ultrasound imaging. With the unprecedented capability of sensing tissue physiological properties with minimal invasion and high resolution deep inside our body, this technology can potentially enable critical applications in both basic research and clinical practice.

## Introduction

Sensing the physiological properties of soft biological tissues is important to understand the development of tissues and help diagnose and treat diseases. The biomechanical and biochemical physiological properties, such as electrophysiologic, metabolism, circulation, thermal properties, and organ mechanics, across a variety of developmental stages in organs and organisms are key to understanding the role of various forces in shaping life (1) associated with growth, aging, regeneration, and wound healing (2, 3). Moreover, the biomechanical and biochemical properties of tissues and organs also have a strong correlation with the development of diseases (4, 5). Sensing the physiological properties of soft tissues deep inside the human body directly and accurately could thus help monitor and understand disease development, as well as provide feedback to therapy.

Methods of using medical imaging tools have been developed to sense the mechanical properties of soft tissues. Optical coherence elastography can sense the elastic property of soft tissues with a spatial resolution at the submillimeter level (6) by combining light transmission and photophysical interactions but has a limited penetration depth of up to several millimeters. Ultrasonic elastography (UE) (7, 8) and magnetic resonance elastography (MRE) (9, 10) have a larger penetration depth (>10 cm), as well as relatively good spatial (0.3 to 0.8 mm) and temporal (several seconds) resolutions, but can only measure elasticity. Recent works on ultrasonic transducers (11–13) attached to the human skin can perform sensing at a spatial resolution of hundreds of micrometers, which is promising for realizing deep (~4 cm) tissue sensing of blood flow and elasticity. Despite these recent advances, there are still limitations of using pure medical imaging to sense advanced physiological properties of tissues. For example, they could only sense the elastic properties of tissues. Moreover, UE typically has a relatively low signal-to-noise ratio (SNR), while MRE cannot be performed on patients with implantable devices and obesity.

On the other hand, flexible and stretchable electronic devices have been used to sense various physiological properties of soft biological tissues on the human skin (4, 5). Passive and active vibration sensors based on resonators on the skin, such as piezoelectric actuators (14) and magnetic actuators based on Lorenz forces (15), can sense the elastic properties of soft tissues, but these methods can only sense physiological signals limited to a few millimeters close to the epidermis, limiting their application for deep tissue sensing. Other flexible sensors implanted inside the organs can sense deep tissue properties but require open surgery for implantation and may potentially cause inflammation (16). For example, existing devices for measuring the pH of tissues, such as electrical impedance spectroscopy (17), are typically wired and rely on endoscope delivery, which is more invasive, and lack the mobility to be easily relocated. Ingestible robotic capsule endoscopes (18–20) with various active locomotion have been shown to sense the physiological properties of the gut in the gastrointestinal (GI) tract. However, they typically have a larger size at the centimeter scale due to the difficulty of scaling down power and communication modules, which limits their access to narrow areas with collapsed tissues and makes them potentially lead to obstruction (19).

Here, we propose a framework combining wireless soft robots and medical imaging to sense advanced physiological properties of tissues, including adhesion, pH, and viscoelasticity deep inside the organs. The millimeter-scale soft robots, actuated by magnetic fields remotely, can attach to and detach from the surface of soft tissues in a controlled manner and interact with soft tissues using their static and dynamic body shapes while being monitored and tracked by medical imaging, such as ultrasound and x-ray medical imaging. Compared with previously reported implanted electronic sensors (14, 21), magnetic microdevices (22, 23), and capsule endoscopes (18–20) for sensing the properties of tissues (20, 21), the untethered multimodal locomotion capability of our wireless soft robot could enable access to enclosed and confined spaces with minimal invasion. Our method could potentially sense the physiological properties of tissues, such as adhesion, pH, and viscoelasticity, which are challenging to be sensed using conventional medical imaging tools, electronic sensors, and other existing medical devices.

## Results

### Sensing mechanism

**Fig. 1:**
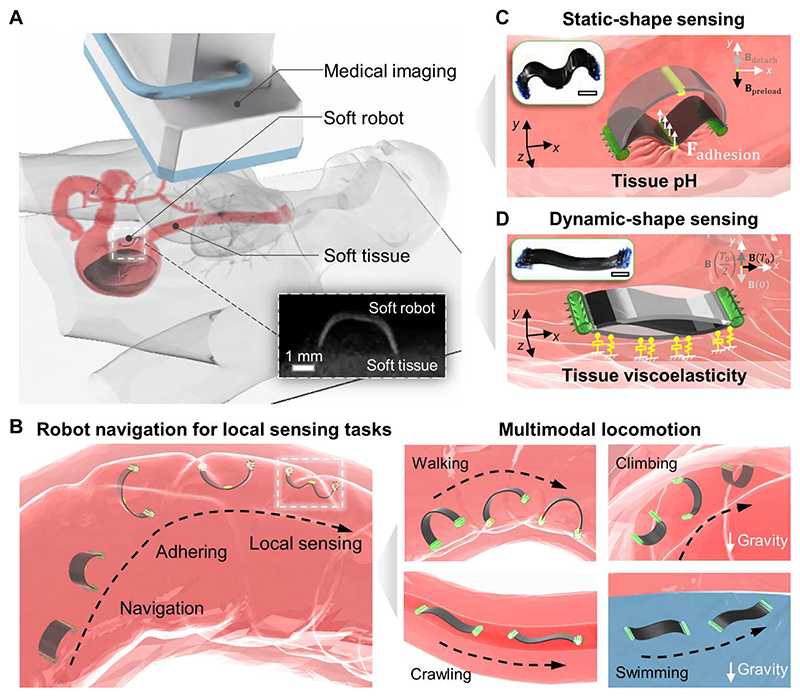
Concept of in situ sensing the physiological properties of soft tissues by a wireless miniature soft robot. (**A**) Concept of wireless miniature soft robots for sensing soft tissue properties in the gastrointestinal (GI) tract. The inner x-ray image presents an example of the visualized robot body and porcine small intestine tissue. (**B**) Schematics of the overall sensing mechanism using a wireless miniature soft robot with multimodal locomotion capability. Left: Process of delivering the robot, robot navigation, and performing local sensing task. Right: Multimodal locomotion modes of the robot, including walking and climbing on tissue surfaces, crawling in tubular channels, and swimming in fluids. (**C**) Sensing soft tissue properties by static robot-tissue interactions, showcased by sensing tissue adhesion and pH values. The subplot illustrates the real robot. (**D**) Sensing tissue properties by dynamic robot-tissue interactions showcased by sensing tissue viscoelastic property. The subplot shows the real robot. In all figures, scale bars are 1 mm.

Our proposed sensing framework is composed of three key components: a wirelessly actuated soft robot; a medical imaging module, such as ultrasound imaging or x-ray fluoroscopy machines, for tracking the robot shape; and the targeted soft tissue (Fig. 1A). The robot soft body shape is precisely controlled by external magnetic fields generated by a customized magnetic actuation system (figs. S1 and S2). As shown in Fig. 1B, the robot has multiple locomotion modes, including walking and climbing on soft tissue surfaces, crawling in narrow crevices, and swimming inside liquids, to navigate through complex terrains and attach to soft tissue surfaces by a bioadhesive patch bonded to the robot body, which is controlled by different external magnetic fields (24). Two types of robot-tissue interaction are reported, which are static and dynamic interactions. In the static interaction, the robot body interacts with the soft tissue in a static manner, where the damping force is negligible, as showcased with a buckling-based motion in Fig. 1C. In the dynamic interaction, the robot constantly experiences a timevarying magnetic field, resulting in a dynamic deformation of the tissue surface, which depends on both the magnitude and frequency of the external magnetic fields. Figure 1D shows that the robot can attach its whole body to the soft tissue surface and perform an undulating motion.

**Fig. 2:**
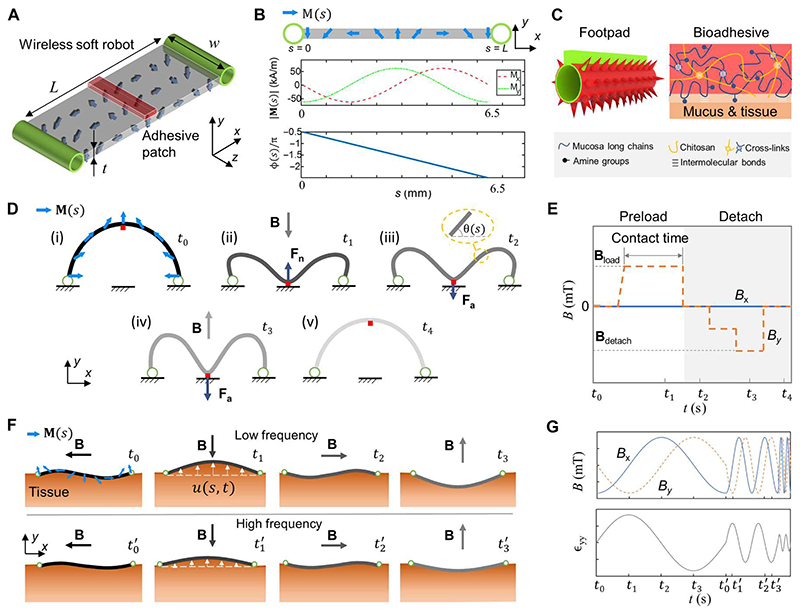
Mechanism of sensing the physiological properties of soft tissues in situ by a wireless miniature soft robot. (**A**) Schematics of the robot design for sensing robot-tissue adhesion. The size of the adhesive patch could be adjusted during fabrication to allow two different attaching modes [partial contact in (D) or full contact in (F)] when contacting soft tissues. The finalized robot size is 6.5 mm × 2 mm × 15 mm *(L* × *w* × *t*). (**B**) Magnetization profile of the soft robot. The blue arrows indicate the magnetization profile **M**(s) of the robot body. (**C**) Robot footpad design with microspikes and chitosan-based bioadhesives (27) for multimodal locomotion. (**D**) Schematics of the buckling-based motion of the robot when interacting with soft tissues for adhesion sensing. (**E**) Time-varying magnetic field for inducing the buckling motion of the robot when interacting with soft tissues. **B**_load_ denotes the magnetic field applied to load the adhesive patch into contact with the substrate. **B**_detach_ represents the magnetic field required to detach the adhesive patch from the substrate. The magnetic fields at time stamps *t*_1_ to *t*_4_ are corresponding to those shown in (D). (**F**) Illustration of the deformation of the robot and tissue in a period when the robot is actuated by a rotating magnetic field of the constant magnitude at various frequencies (top, low frequency; bottom, high frequency). (**G**) Magnetic field waveforms for inducing the dynamic shapes of the robot. The time frames correspond to their counterparts in (F). *ε_yy_* represents the material strain at the location of *s* = 3.25 mm at the robot-material interface.

To show the fundamental mechanisms of sensing soft tissue physiological properties, we develop a wireless soft millirobot by bonding bioadhesive patches on the robot body. Our control strategies allow the robot to interact with soft tissues for in situ sensing, where we implemented our previously reported switchable tissue adhesion mechanism (24) on this robot. As shown in Fig. 2A, the robot has a magnetoelastic soft body, two ring-shaped footpads, and an adhesive patch bonded to the body surface. The robot (length, *L;* width, *w;* thickness, *t*) has magnetization magnitude and phase profiles **M**(s) and φ(*s*) (*s* ∈ [0, *L*], *s*: material coordinate) shown in Fig. 2B, which allows it to realize desired multimodal locomotion, including climbing, crawling, and swimming (24). The robot foot-pads have microspikes coated with hydrogel and tough bioadhesives made of chitosan, as shown in Fig. 2C, so that it can attach to and detach from tissue surfaces controlled by external magnetic fields. The conical microspikes ensure large friction on tissue surfaces, while the biocompatible chitosan-based bioadhesive, capable of cross-linking with the mucus layer, provides large adhesion (24).

Two fundamental mechanisms have been proposed for sensing the physiological properties of tissues in situ. First, the robot can use buckling-based static shapes to sense robot-tissue adhesion (Fig. 2D). Such adhesion measurement can also be used for sensing various mechanical properties of tissues, such as pH and temperature, by using stimuli-responsive adhesives (25). A remote magnetic field (Fig. 2E) is controlled to induce the buckling motion by the distributed magnetic torque along the robot body, which will be discussed in detail in the next section. The fundamental principle is that when the robot’s two footpads attach to a soft tissue surface (Fig. 2D, i), a magnetic field **B** perpendicular to the tissue surface in the negative *y* direction will induce the deformation of the robot body toward the tissue surface (Fig. 2D, ii). The adhesive patch will be loaded to the tissue surface due to a buckling effect (26). After removing the magnetic field, the robot will maintain the buckled shape due to the patch-tissue adhesion (Fig. 2D, iii). Gradually increasing the magnetic field in the positive *y* direction (Fig. 2D, iv) will result in a distributed magnetic torque to detach the adhesive patch from the tissue surface once the **B** field reaches a threshold value (Fig. 2D, v). Moreover, the patch-tissue adhesion can be estimated using the robot body shape and the input magnetic field based on a magnetoelastic model using the Euler-Bernoulli Beam theory (see the “Estimating robot-tissue adhesion” section in note S1). As illustrated in Fig. 2D, the static shape of the robot body is described by θ(*s*) and $\frac{\partial \theta }{\partial s}$, which are the slope angle and curvature of the robot body at a specific location *s* ∈ [0, *L*], respectively. The robot shape data can be obtained by optical imaging in vitro and medical imaging in vivo, while the input magnetic field can be obtained from the calibrated magnetic actuation system (fig. S1).

In addition, our robot can also adhere to and dynamically interact with soft tissues to sense their viscoelastic properties under a periodic magnetic field waveform (Fig. 2F). For example, Fig. 2G shows rotating magnetic fields with a constant magnitude at various frequencies. The tissue viscoelastic properties can be estimated from the tissue normal strain $\varepsilon_{yy}^{t}$ (or the tissue normal displacement $u_{y}^{t}$) and the tissue normal stress $\sigma_{yy}^{t}$ at the robot-tissue interface. At the robot-tissue interface, we have $\sigma_{yy}^{t}=-\sigma_{yy}^{r}$ and $u_{y}^{t}=u_{y}^{r}$, where $\sigma_{yy}^{r}$ and $u_{y}^{r}$ are the robot’s normal stress and displacement, respectively. We can further use the dynamic shape of the robot to approximate the tissue strain and stress using a magnetoelastic model. Given a specific robot, we assume that the normal stress of the soft tissue is proportional to the external magnetic field with a scaling factor *k_y_* assuming small deflections. When subjected to a rotating magnetic field with a frequency of *f* = *ω*/2π, $\sigma_{yy}^{r}$, can be estimated from the magnetic field given by $\sigma_{yy}^{r}=k_{y}\left[M(s),K_{r}\right]B(\omega t)$, where *k_y_* is a function of the bulk modulus *K_r_* and the magnetization profile *M*(*s*) of the robot, independent of the external magnetic field and the soft tissue properties. Therefore, if we perform the frequency sweeping for the function $\varepsilon_{yy}^{t}(\omega t)/B_{y}(\omega t)$ by varying *ω*, we can estimate the storage and loss moduli of the tissue *E’* and *E”* using the low- and high-frequency parts of the frequency response described in the “Estimating tissue viscoelastic properties”section in note S2.

### Sensing robot-tissue adhesion using static robot-body shapes

**Fig. 3:**
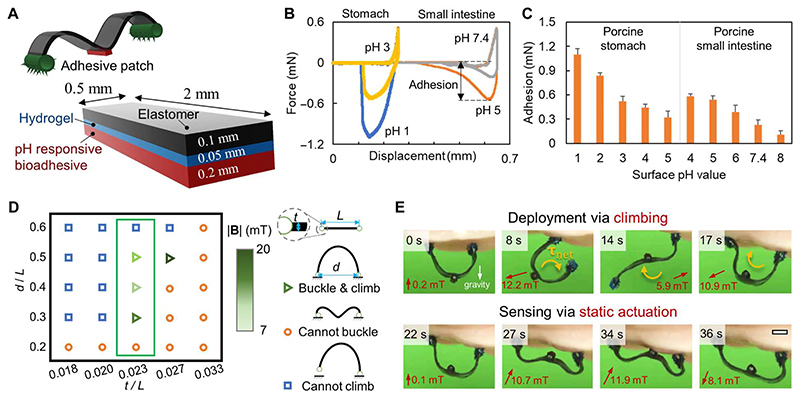
Sensing the adhesion and pH of soft tissues using a wireless miniature soft robot integrated with a pH-responsive adhesive patch. (**A**) Design of the soft robot with a pH-responsive bioadhesive patch. (**B**) Normal force between the adhesive patch and the soft tissues as a function of the displacement. The adhesion is quantified using the maximum pull-off force (negative normal force) as denoted in the plot. Soft tissue: porcine stomach and porcine small intestine. (**C**) pH-responsive properties of the adhesive patch on porcine stomach and small intestine tissues. (**D**) Feasible robot thickness-to-length ratio (TLR) for realizing both the climbing locomotion and the buckling-based deployment. *t*, *L*, and *d* are the robot body thickness, body length, and the distance between the footpads when buckling, respectively. The selected robot geometry design is marked by the green box. A small *d/L* disables the buckling due to the large curvature of the robot body and consequently a large bending stiffness, while a large *d/L* hampers the climbing mobility because of the insufficient net magnetic torque. (**E**) Climbing-based deployment of the robot to the targeted position and the buckling-based sensing of the robot adhesive patch to the soft tissue surfaces. Scale bar, 1 mm. In all figures, the error bar represents the SD for *n* = 5 measurements. The measurements involved a pH-responsive bioadhesive patch per sample, with *n* indicating the number of samples.

In Fig. 3 and movie S2, we present the robot design, characterization, sensing mechanism, and validation results for sensing robot-tissue adhesion and, therein, the tissue pH values at a specific location. A 2 mm × 0.5 mm × 0.35 mm adhesive patch coated with pH-sensitive bioadhesives is bonded at the middle of the robot body (Fig. 3A). The patch is composed of three layers: a layer of elastomer (thickness: 0.1 mm), a thin layer of coated hydrogel (thickness: 0.05 mm), and a layer of pH-sensitive bio-adhesive (thickness: 0.2 mm). The hydrogel layer serves as a dissipative matrix material for tough adhesives (27) as well as a bonding material between the elastomer patch and bioadhesive. The pH-sensitive bioadhesive layer combines both the chitosan-based bioadhesive for tough adhesion to mucus-covered tissues via cross-linking (27) and the catechol-boronate–based hydrogel adhesive for pH-responsive adhesion via amine-borate complexation (see the “Fabrication of the adhesive patch for sensing adhesion and pH” section in Materials and Methods) (28, 29).

We quantify the pH-sensitive adhesion to tissue surfaces of the adhesive patch in Fig. 3 (B and C). Figure 3B compares the adhesion of the adhesive patch on ex vivo porcine stomach and small intestine tissue surfaces with different pH values. Standard phosphate-buffered saline (PBS) buffer solutions with different pH values are added to the same tissue surfaces to achieve the desired pH values, validated by a pH indicator. With a preload of 0.5 mN in a customized adhesion measurement setup (fig. S3 and movie S2), we perform a systematic measurement of the patch-tissue adhesion. The adhesive patch shows adhesion of 1.08 ± 0.07 mN on an acidic porcine stomach surface with pH 1 but only 0.54 ± 0.06 mN on the same porcine stomach surface with pH 3. Furthermore, the adhesive patch shows the adhesion of 0.56 ± 0.04 mN on the porcine small intestine surface with pH 5, while only 0.23 ± 0.09 mN on the same porcine small intestine surface with pH 7.4.

In Fig. 3C, we further prove that the patch-tissue adhesion is almost linearly dependent on the pH of the tissue surface so that the patch-tissue adhesion can be used to sense pH assuming similar surface conditions. First, the pH-responsive properties of the adhesive patch can be designed to sense a wide range of pH values. For example, the adhesion decreases from 1.08 ± 0.07 to 0.31 ± 0.12 mN on the porcine stomach tissue surfaces when the pH increases from 1 to 5. This pH range can cover most of the possible cases in the stomach. For example, the average pH is about 5.4 ± 2.1 in patients with gastric cancer, 3.0 ± 2.2 for gastritis, 2.4 ± 1.9 for gastric ulcers, 1.3 ± 0.6 for duodenal ulcers, and 1.7 ± 0.2 for normal subjects (30). Meanwhile, the adhesion decreases from 0.58 ± 0.03 to 0.11 ± 0.05 mN on porcine small intestine tissue surfaces when a pH increases from 4 to 8. In addition, our pH-responsive adhesive patch shows a comparable sensitivity of 1 pH unit like the commercial pH-indicator strips pH 2.0 to 9.0 (MQuant 1.09502, Sigma-Aldrich Inc.) with a sensing range from 1 to 8.

Figure 3D shows the design of the robot for achieving both desired climbing locomotion and the adhesion sensing function. To achieve both climbing locomotion and deployment for sensing, we optimize the robot body design with a key design parameter—the thickness-to-length ratio (TLR), which compromises the climbing locomotion and the buckling-based attachment to tissues. With a relatively small TLR, the robot can be deployed with a buckling motion easily due to a relatively small bending stiffness, but the robot has degraded climbing locomotion due to insufficient net magnetic torque (fig. S4). We perform a systematic parameter sweeping of the TLR from 0.018 to 0.033 (body thickness: 0.15 mm) to optimize the TLR for both the robot climbing locomotion and the buckling-based attachment to tissues. We find that a TLR of 0.023 (body length: 6.5 mm) allows a sufficient climbing ability with a wide range of reachability quantified by the ratio of the distance between the footpads to the length of the robot body (0.3 to 0.5) while using a relatively small actuation magnetic field (<20 mT). The robot with a TLR of 0.023 also allows easy deployment, as showcased in Fig. 3E and movie S1.

**Fig. 4:**
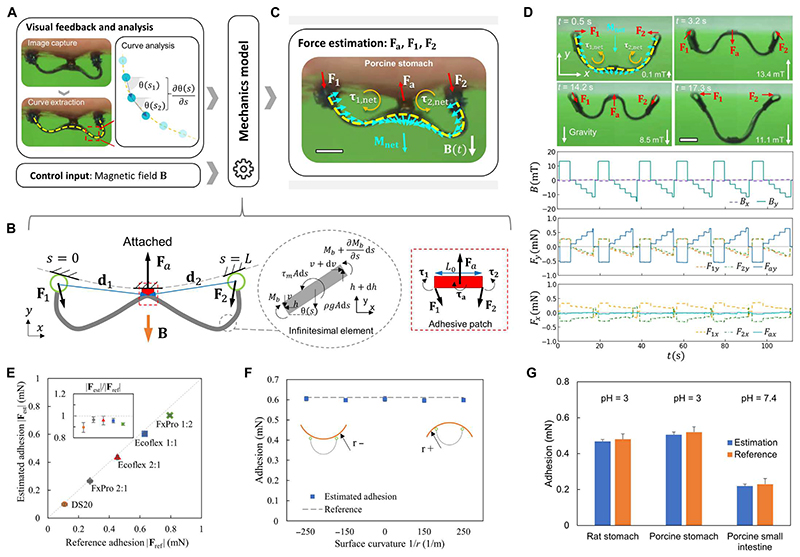
Quantification of the performance of sensing robot-tissue adhesion and therein the pH. (**A**) Schematics of the overall sensing mechanism. The robot shape is assumed to be known and described by functions θ(*s*) and $\frac{\partial \theta (s)}{\partial s}$. The magnetic field input is assumed to be known as **B**(*t*). (**B**) Schematics of the robot-tissue interaction model. (**C**) Schematics of the distributed torque and force applied to the robot body. (**D**) Top: Snapshots of the robot at rest, the robot loaded for attachment, the robot to be detached, and the robot just detached (movie S2). Bottom: Magnetic field input signals and the estimated forces. The proposed method yields consistent adhesion estimation results for over five repetitions with an average relative SD of 4.4%. Synthetic substrate material: FxPro 1:2 (Dragon Skin FX Pro/1 Silicone Rubber; mixture ratio, part A:part B = 1:2 by weight, Smooth-On Inc.). (**E**) Comparison of the estimated and real robot-tissue adhesion on synthetic materials (see “Preparation of synthetic materials for adhesion measurements”section in Materials and Methods). (**F**) Estimated adhesion on curved surfaces of synthetic materials. Estimated adhesion lies within the range of 0.596 to 0.606 mN compared to the measured adhesion of 0.612 ± 0.020 mN. *r* is the curvature radius. Material: Ecoflex 0030 silicone rubber with a weight ratio of 1:1 (Smooth-On Inc.). (**G**) Estimated robot-tissue adhesion on different animal soft tissue surfaces. In all figures, scale bars are 1 mm. Error bars indicate the SD for *n* = 5 measurements. For the estimation data, the measurements involved a robot per sample, with *n* indicating the number of samples. For the reference data, *n* is the number of samples measured by the setup in fig. S3.

Figure 4A shows the schematics of estimating the robot-tissue adhesion using a magnetoelastic model. Given the robot static shapes parameterized by θ(*s*), $\frac{\partial \theta (s)}{\partial s}$ and the external magnetic field **B**, we can estimate the patch-tissue adhesion. Figure 4B illustrates the free-body diagram of the robot under a “pinned-fixed-pinned” boundary condition, where the adhesion **F**_a_ between the adhesive patch and the tissue surface is balanced by the reaction forces **F_1_** and **F_2_** in addition to the robot body weight. As shown in Fig. 4C, **F_1_**, **F_2_**, and **F_a_** can be estimated by solving the moment and force balance equations based on the Euler-Bernoulli beam theory (see “Estimating robot-tissue adhesion” section in note S1). As an example, Fig. 4D illustrates the sensing process of the robot-material adhesion **F**_a_ on a substrate surface of Ecoflex-0030 silicone rubber (1:1 weight ratio). In this process, the robot is first loaded for attachment with an external magnetic field of 13.4 mT for 5 s in the +*y* axis. Then, an external magnetic field in the — *y* axis is applied from 0 mT with a magnitude interval of 2.7 mT and a time interval of 2.5 s until the robot is detached from the surface. Note that our method yields consistent adhesion estimation results for over five repetitions with an average relative SD of 4.4%, as shown in fig. S5.

To further verify our sensing method, we compare the estimated adhesion using the soft robot on various synthetic substrate surfaces with the adhesion measured by a high-precision force sensor in a customized adhesion measurement setup (fig. S3). Figure 4E compares the estimated adhesion and the reference adhesion for synthetic surfaces with various adhesion properties. For the adhesion range from 0.13 ± 0.02 to 0.82 ± 0.02 mN, the estimated results from the proposed sensing method match the reference adhesion with relative errors all less than 12%, as shown in Fig. 4E and movie S2. The proposed method is also applicable to a substrate surface with relatively large curvatures. We perform the sensing experiments on the convex, flat, and concave surfaces with a curvature of –250, –150, 0, 150, and 250 m^−1^ covered by the 2-mm-thick Ecoflex-0030 silicone rubber sheets (1:1 weight ratio). The relative errors between the estimated adhesion on curved surfaces and the measured force are less than 2.6% (Fig. 4F). In summary, the proposed sensing method can estimate the robot-substrate adhesion with a relative error less than 20% for a wide range of adhesion from 0.13 ± 0.02 to 0.82 ± 0.02 mN and various curvatures from –250 to 250 m^−1^.

To further quantify the performance of sensing robot-tissue adhesion and therein the pH values, we use the robot to sense adhesion on various tissue surfaces with different pH values. Figure 4G compares the estimated adhesion by the proposed method and the reference adhesion measured by a high-precision force sensor (fig. S3) on tissues, including rat stomach, porcine stomach, and porcine small intestine (see fig. S6 for more detailed comparisons for the same tissues covered by PBS solutions of various pH values). The relative errors in adhesion between the estimation and the measured values for all three tissues are less than 10%. Therefore, the proposed method could potentially enable minimally invasive sensing of the tissue adhesion and pH at a hard-to-reach spot inside the human body with the aid of medical imaging devices.

### Sensing viscoelasticity of soft tissues using dynamic robot-body motion

**Fig. 5:**
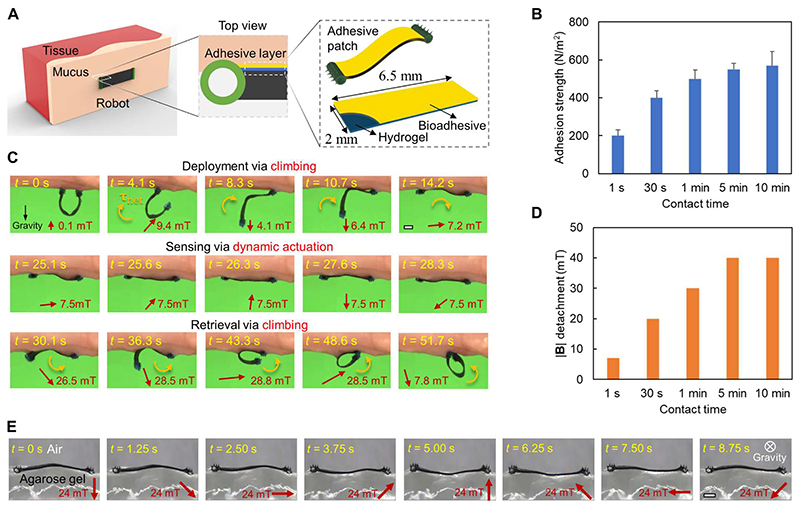
Sensing the viscoelasticity of soft tissues using a wireless soft robot integrated with a bioadhesive patch. (**A**) Bioadhesive patch design. The adhesive patch is made of a hydrogel layer coated with bioadhesives. (**B**) Adhesion between the robot adhesive patch and the soft tissues as a function of the contact time. The soft tissue is from the porcine small intestine. Error bars indicate the SD for *n* = 5 measurements. The measurements involved a robot adhesive patch per sample, with *n* indicating the number of samples. (**C**) Video snapshots (movie S1) of the deployment, sensing, and retrieval process of the soft robot on the porcine small intestine surfaces. (**D**) Magnitude of the applied rotating magnetic field for detaching the adhesive patch from soft tissues as a function of the contact time. The soft tissue is from the porcine small intestine. (**E**) Video snapshots (movie S3) of the robot dynamic shape when interacting with the agarose gel (0.3 wt %) under a rotating magnetic field with a magnitude of 24 mT and a frequency of 0.1 Hz. In all figures, scale bars are 1 mm.

In Fig. 5, we present the design of the soft robot to sense tissue viscoelastic properties with dynamic robot-body motion. To maintain the robot-tissue attachment during the dynamic interactions, a 6.5 mm × 2 mm adhesive patch is bonded to the robot body (Fig. 5A). The adhesive patch is fabricated by coating the robot body with hydrogel and bioadhesive sequentially with a total thickness of about 0.05 mm (see the “Fabrication of the adhesive patch for sensing tissue viscoelasticity” section in Materials and Methods). The adhesive layer bonds the robot and tissue surfaces together while having a minimal effect on the robot-tissue dynamic interactions. We further show that the adhesion strength between the robot and tissue surfaces can be adjusted by the contact time in Fig. 5B. The adhesion between the bioadhesive patch and the porcine small intestine tissue is a function of the contact time, characterized by a customized setup with a preload of 0.1 mN (fig. S3). The bonding process involves the intermolecular interaction and cross-linking between the bioadhesive and the mucus, which is time dependent (29). When the bonding is sufficiently formed (after 1 min), the adhesion per unit area is around 509 ± 47 N/m^2^ and large enough to maintain the robot-tissue attachment. In addition, the adhesion per unit area is still within 574 ± 83 N/m^2^ as the contact time reaches 10 min, enabling the on-demand detachment under magnetic actuation.

Figure 5C shows the robot attachment, sensing, and detachment behaviors for sensing tissue viscoelasticity. First, the robot is deployed and adheres its bioadhesive side to the targeted tissue area with the whole-body torque generated by the nonzero net magnetic torque **τ_net_**. Then, the robot is actuated to sense tissue viscoelasticity under a rotating magnetic field (magnitude: 7.5 mT). When the sensing process is completed, the robot body is detached from the tissue surface under a magnetic field with a relatively large magnitude (~26 mT). Figure 5D shows the minimum magnetic field needed for the detachment as a function of the contact time when the robot is attached to the porcine small intestine tissue. A more detailed illustration of the robot dynamic motion under a rotating magnetic field is presented in Fig. 5E and movie S3, where the robot undulates when attaching to an agarose gel [0.3 weight % (wt %)] back layer under a rotating magnetic field (24 mT, 0.1 Hz).

**Fig. 6:**
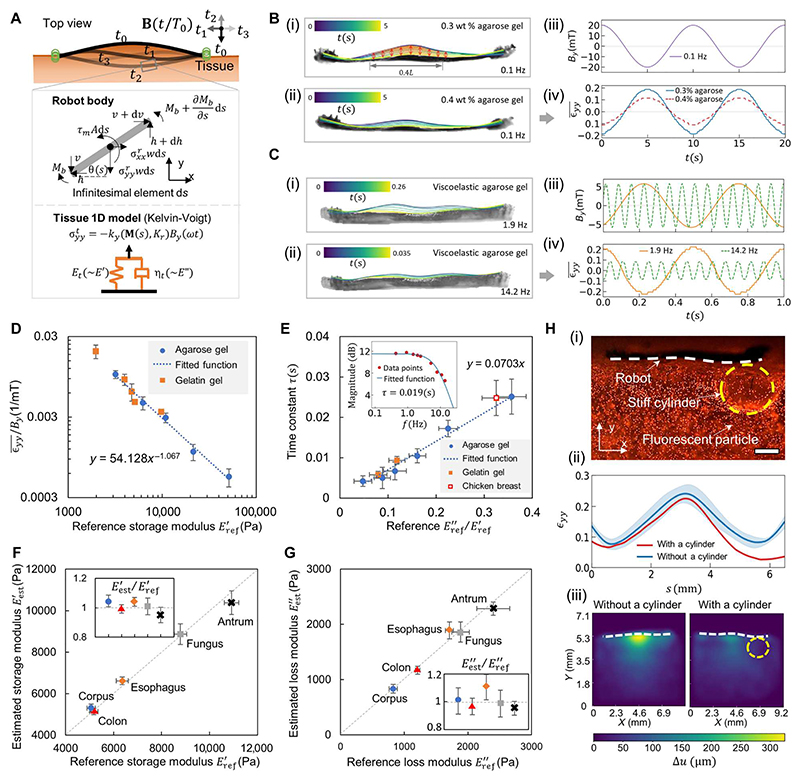
Quantification of the performance of sensing viscoelasticity. (**A**) Illustration of the mechanic model for the dynamic robot-material interaction. (**B**) (i and ii) Robot dynamic shape when interacting with different synthetic materials. Material: 0.3 and 0.4 wt % agarose gel with *E’* = 3.2 ± 0.3 and 6.2 ± 0.5 kPa, respectively. (iii and iv) Applied magnetic field waveform and the measured normal strain $\bar{\varepsilon_{yy}}$ at the robot-material boundary. $\bar{\varepsilon_{yy}}=\int_{0.3L}^{0.7L}\varepsilon_{yy}(s)ds/0.4L(L=6.5\text{mm})$, as the middle part of the robot body has the largest signal-to-noise ratio (SNR) (see fig. S5). (**C**) (i and ii) Robot dynamic shape subject to rotating magnetic fields of different frequencies. (iii and iv) *B_y_* and $\bar{\varepsilon_{yy}}$ at the robot-material boundary. Material: 70-0.3 agarose-based gel (weight ratio, sucrose:water:agarose = 70:30:0.3) with *E”* = 439.1 ± 46.8 Pa. (**D**) Correlation between $\bar{\varepsilon_{yy}}/B_{y}$ and the reference storage modulus $E_{\text{ref}\;}^{\prime }$ of different synthetic gels. (**E**) Correlation between the measured time constant τ and the ratio of the characterized loss modulus $E_{\text{ref}}^{\prime \prime }$ and storage modulus $E_{\text{ref}}^{\prime }$. Subplot: frequency response of the robot normal deformation [magnitude: $20\log\left(\bar{\varepsilon_{yy}}/B_{y}\right)$]. (**F** and **G**) Comparison of the estimated and real (F) *E’* and (G) *E”* of different porcine tissues. (**H**) Sensing simulated disease spots spatially. (I) Fluorescence image of a tested sample. A stiff cylinder of 1.0 wt% agarose gel (*E’* = 51.2 ± 0.6 kPa; diameter, 2 mm) is embedded inside the 0.3 wt % agarose gel (*E* = 3.2 ± 0.3 kPa). (ii) Comparison of the robot deformation with and without a cylinder. (iii) Comparison of the material maximum displacement distribution with and without a cylinder. Scale bar, 1 mm. In all figures, error bars represent the SD of *n* = 5 measurements. For the estimation data, the measurements involved a robot per sample, with *n* indicating the number of samples. For the reference data, *n* is the number of samples measured by the rheometer (Discovery HR-2, TA Instruments).

We further demonstrate the method to estimate the tissue storage modulus *E’* and the loss modulus *E”* using a frequency sweeping method based on the mechanical model in Fig. 6A. On the one hand, *E*′is estimated under a low-frequency (0.1 Hz) rotating magnetic field. In this case, the robot deformation magnitude is only dependent on *E’* and the effect of *E”* is negligible. As illustrated in Fig. 6B, the robot shows distinct deformation magnitudes when interacting with materials of different *E’* upon applying the same low-frequency magnetic field (20 mT, 0.1 Hz). The average material strain $\bar{\varepsilon_{yy}}$ at the robot-material interface, calculated by $\bar{\varepsilon_{yy}}=\int_{0.3L}^{0.7L}\varepsilon_{yy}(s)ds/0.4L$ (*L* = 6.5 mm; see Fig. 6B, i, and fig. S7), varies significantly with the amplitude of 0.19 and 0.11 for *E’* = 3.2 ± 0.3 kPa and 6.2 ± 0.5 kPa, respectively.

On the other hand, *E”* is estimated from the robot deformation magnitude change by sweeping the rotating magnetic field at different frequencies. As shown in Fig. 6C, the robot deforms less for the same material when the actuation frequency increases, and the $\bar{\varepsilon_{yy}}$ amplitude drops from 0.23 to 0.08 under the actuation frequency of 1.9 and 14.2 Hz. In our method, the scaling factors *k* and *k_τ_*, independent of materials, are first calibrated using synthetic materials to obtain the function $E^{\prime }\left(\bar{\varepsilon_{yy}}/B_{y}\right)$ and $E^{\prime \prime }\left(\bar{\varepsilon_{yy}}/B_{y}\right)$, which are further used to estimate *E’* and *E*” of tissues (see the “Estimating tissue viscoelastic properties”section in note S2). First, *E’* $E^{\prime }\left(\bar{\varepsilon_{yy}}/B_{y}\right)$ is estimated under the actuation frequency of 0.1 Hz. In Fig. 6D, we use the agarose and gelatin gels as the training and validation materials to obtain the fitted function $\bar{\varepsilon_{yy}}/B_{\text{y}}=54.128{\left(E^{\prime }\right)}^{-1.067}$. In this function, *k* is calibrated as 54.128 Pa/mT, and the index value –1.067 is close to –1, which validates that *E*” has a negligible effect on the estimation of *E’* under the 0.1-Hz actuation. It is notable that the *E’* of the experiment synthetic materials ranges from 1.9 ± 0.2 kPa to 51.2 ± 0.6 kPa, which covers the stiffness range of most soft tissues (31). After *E’* is estimated, the frequency sweeping method is used to compute the time constant τ, as shown in the subplot of Fig. 6E. Further, *k_τ_*, the ratio of τ to *E’*/*E”*, is calibrated with the agarose-based viscoelastic gel and further validated by the gelatin-based gel and chicken breast tissue (*k*_τ_ = 0.0703 s; Fig. 6E), through which *E*” is obtained on the basis of the estimated *E’* and τ. Last, the healthy porcine GI tissues are adopted to test the viscoelasticity sensing method ex vivo. Figure 6 (F and G) shows that the estimated *E’* and *E*” of the tissues match the reference moduli characterized by the rheometer. The relative errors are within 8% for *E*′ and 12% for *E*”, as shown in the subplots of Fig. 6 (F and G). Notably, the degree of robot deformation can be controlled by adjusting the magnetic field. This helps minimize the effect of sample boundary conditions on the robot deformation, particularly when the material thickness is small (see fig. S8), and ε_yy_ in the robot coordinate system is adopted for the calibration process (see fig. S9).

Our robot also has the potential to achieve distributed sensing due to the continuous stress imposed on the contact interface along the robot body. The method can be potentially used for disease location sensing during its development, such as cystic fibrosis in lungs (32). As illustrated in Fig. 6H (i) and movie S3, the robot contacts the stiff cylinder and is actuated by the rotating magnetic field, during which the robot deformation difference with and without a stiff cylinder can indicate the cylinder location in Fig. 6H (ii). Correspondingly, the material deforms less at the cylinder location (Fig. 6H, iii). In fig. S10, the location and diameter of the cylinder are adjusted to simulate different occasions, and the corresponding robot and material deformation are shown in figs. S11 and S12. By analyzing the robot deformation, the cylinder location and its influence on the surrounding area can be quantified (fig. S13).

### Sensing soft tissue mechanics ex vivo for organs with diseases

**Fig. 7:**
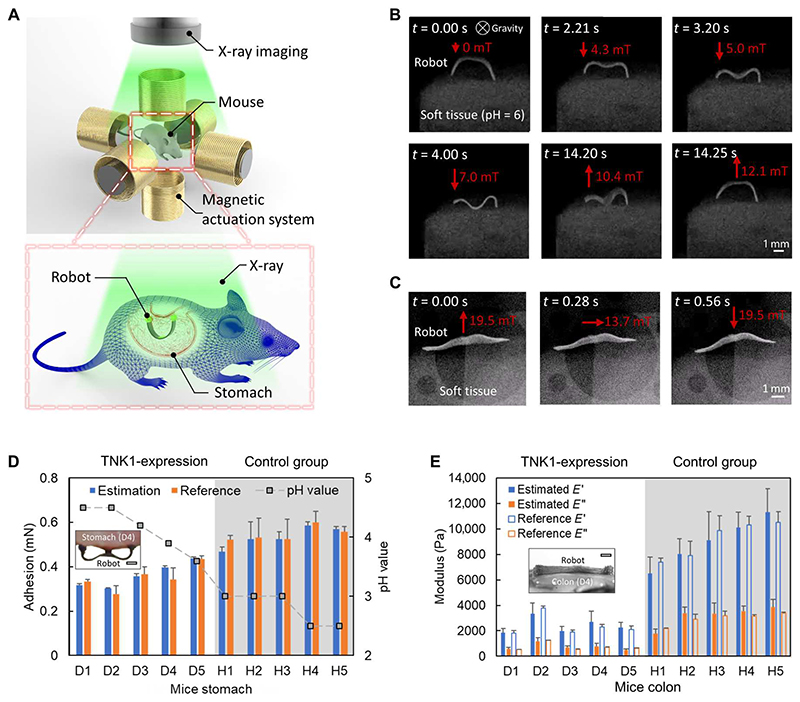
Demonstration of measuring the physiological properties of soft tissues in an ex vivo intestinal disease model using x-ray medical imaging. (**A**) Schematics of sensing the tissue physiological properties in ex vivo disease model under x-ray medical imaging. (**B**) Video snapshots (movie S4) of sensing the pH of the tissue by the robot with the pH-responsive bioadhesive patch under x-ray cabinet imaging. (**C**) Video snapshots (movie S5) of sensing the viscoelastic properties of the tissue by a soft robot with the bioadhesive patch attached to one side of the whole robot body visualized by x-ray cabinet imaging. (**D**) Sensed adhesion between the soft robot and stomach tissue of mice with and without the thirty-eight–negative kinase 1 (TNK1) expression–related disease (34). The subfigure shows sensing adhesion on mice stomach tissues (D4) ex vivo. (**E**) Sensed viscoelastic properties of colon tissue of mice with and without the TNK1 expression–related disease. The subfigure indicates sensing viscoelasticity on mice colon tissues (D4) ex vivo. In all figures, error bars represent the SD. For the estimation data, *n* = 3 tissue samples were prepared, with a robot attached per sample. For the reference data, *n* = 5 tissue samples were measured by the customized setup (fig. S3) for adhesion or the rheometer (Discovery HR-2, TA Instruments) for viscoelastic properties. Scale bars, 1 mm.

To show the potential of using our robot to monitor and understand the biomechanics of soft tissues with diseases, we present ex vivo sensing tissue pH and viscoelastic properties of soft tissues using our robot together with x-ray imaging in Fig. 7 and movies S4 and S5. The robot is first deployed into the diseased area of the mouse models and then actuated by the external magnetic field to implement the sensing functions, as shown in Fig. 7A. An x-ray cabinet imaging tool tracks the robot and surrounding soft tissues during the sensing processes. Figure 7 (B and C) shows that the static and dynamic robot-body shapes can be distinguished from the soft tissues in the x-ray images. For dynamic shape-based sensing, this framerate can allow sensing the storage modulus *E*’ of soft materials and the loss modulus *E*” of the soft material with a cutoff frequency up to 7 Hz based on the Nyquist sampling criterion. The x-ray imaging device has a framerate of 30 frames per second (FPS), which is sufficiently fast for imaging robot-body dynamic motion with a frequency of less than 10 Hz. Notably, the x-ray radiation dose in our experiment is under 410 μSv/hour (see the “x-ray medical imaging” section in Materials and Methods), which is safe for live animal experiments (33). In addition, we also show that ultrasound imaging can be used for tracking the robot body shape to sense tissue viscoelastic properties with a framerate of up to 30 FPS (fig. S14).

Further, mice with forced expression of thirty-eight–negative kinase 1 (TNK1), which exhibit impaired intestinal barrier (34), and healthy wild-type littermates are used to showcase the proposed methods for sensing pH and viscoelasticity to diagnose diseases. Regulation of apoptosis and immunomodulatory functions have been ascribed to TNK1, which is a promising target during multi-organ dysfunction syndrome to prevent damage in several organs, especially the gut. Because of the TNK1 expression, the normal intestinal architecture is perturbed. In addition, the basal pH in the stomach of the TNK1-expression mouse is higher than that of a mouse from the healthy control group (fig. S15) since the diseased mice are fasted because of abnormal intestinal activities (34).

Figure 7D compares the robot-tissue adhesion estimated by the proposed method, the reference data measured by the high-precision force sensor, and the pH value measured by the pH indicator of the mice stomach tissues with (H1 to H5) and without a TNK1 expression–related intestinal disorder/disease (D1 to D5). Despite the deviations among individuals from the same group, the TNK1-expression disease shows high relevance with the estimated adhesion results, which also matches the reference results and the corresponding pH values. The proposed adhesion sensing method shows its ability to indicate the difference in adhesion or pH of the ex vivo tissues, with 0.32 ± 0.01 mN to 0.44 ± 0.01 mN for TNK1 expression–diseased mice compared to 0.47 ± 0.02 mN to 0.59 ± 0.02 mN for healthy mice, potentially serving as a minimally invasive tool for detecting diseases.

Figure 7E compares the storage and loss moduli estimated using our method and the reference data characterized by a rheometer of the colon tissues of mice with (H1 to H5) and without a TNK1 expression–related disease (D1 to D5). The estimated storage and loss moduli for the TNK1-expression mice range from 1.9 ± 0.3 to 3.4 ± 0.8 kPa and 0.5 ± 0.1 to 1.2 ± 0.3 kPa, respectively, whereas the estimated storage and loss moduli for the healthy mice are from 6.5 ± 1.3 to 11.3 ± 1.8 kPa and 1.8 ± 0.4 to 3.8 ± 0.6 kPa, respectively. The estimated results are consistent with the reference data measured by the rheometer and can indicate the abnormal viscoelastic properties of the tissues.

## Discussion

We have reported a generic framework to sense the physiological properties of soft tissues using wireless miniature soft robots and medical imaging. A fundamental magnetic-mechanical model is developed to recover the mechanical properties of soft tissues, such as adhesion and viscoelasticity, with the tracked robot shape changes using medical imaging. This framework has been showcased with two example applications. One is a soft robot with an integrated pH-responsive adhesive patch for sensing robot-tissue adhesion and therein the correlated pH values using a buckling motion. The other one is a soft robot with an integrated adhesive patch that can be deployed on soft tissue surfaces and measure the viscoelasticity of soft tissues using a dynamic undulating motion. We have performed systematic experiments on synthetic materials and ex vivo soft tissues and validated the sensing mechanisms by comparing them with direct and high-precision measurements of the synthetic materials and biological soft tissues. Last, we have demonstrated the feasibility of sensing the mechanics of soft tissues in mice with diseases as a tool for diagnostics and monitoring GI tract diseases.

Compared with other implantable magnetic and electric devices where the components are embedded inside the tissues (22, 23), our device can traverse complex soft and wet tissues to provide minimally invasive sensing ability to a wide range of tissue properties. Our robots can bond to the surfaces of soft tissues on demand and sense the advanced physiological properties of soft tissues. The deployment of the robot can be realized by a soft-body self-deformation or using an existing medical device, such as an endoscope. On the other hand, existing miniature robots have only shown simple in vitro sensing of temperature using thermally responsive liquid elastomer materials on a magnetic body (35). Our soft robot–based approach can locally sense the tissue biomechanics in vivo, where the medical imaging signals are amplified by the body shapes, allowing sensing various physiological properties of soft tissues. In addition, our method can potentially achieve a higher SNR compared with UE. The SNR of the basic UE is below 6 (36) and can be improved to up to 14 with the optimization algorithms (37, 38), while the SNR of our method can reach 39 because of the high contrast between the robot and the sensed materials under x-ray imaging (see the “Calculating the signal-to-noise ratio”section in note S3).

The biocompatibility of our soft robot can be ensured by coating a thin layer of biocompatible materials such as polydimethylsiloxane (PDMS) (39) or parylene C (40) to prevent the toxic NdFeB particles from contacting the tissues. We will investigate the biocompatibility before and after coating with the standard cell survival test in the future. In addition, our soft robot–based sensing may be affected by the fluid flow but might be compensated with faster medical imaging and integrating the robot-fluid interaction into the mechanical model, which will be explored in future work. Moreover, despite that the multistep fabrication process can result in a variance of the robot properties (fig. S16), the current experimental results show acceptable deviation in the measured properties of the tissues using five different robots. However, we can always calibrate the robot properties for better accuracy before using the robot for sensing.

Our demonstrated sensing mechanisms can potentially be used for sensing peptic ulcers (41) in the GI tract, where the pH level changes markedly compared with normal tissues, as well as pulmonary fibrosis (42) and cancer (43), where the viscoelastic properties have changed substantially when the disease is developing. It should be noted that it is indeed difficult for our robot to detect a sudden change in the pH value, which generally takes about 30 s to sense the pH with our robot. However, the pH of the surfaces in the GI tract does not change rapidly (44). We can improve the sensing speed by integrating more sensitive bioadhesives, such as optimizing the concentration of the amine groups and borate ester groups in the bioadhesives (28, 29) or increasing the surface area to volume ratio by incorporating microstructures into the adhesive patch design (45), in the future.

The proposed framework of using wirelessly actuated miniature robots to interact with soft tissue surfaces while monitoring their shape deformation using medical imaging thus opens a door for monitoring and understanding tissue biomechanics in vivo during disease development as well as providing feedback information for the applied therapeutic solutions. Our method thus adds unprecedented capabilities to available minimally invasive sensing methods and medical devices, potentially enabling versatile applications in both basic research and clinical practice.

## Materials and Methods

### Fabrication of the soft millirobot

As shown in fig. S17A, the footpads were made of PDMS (Sylgard 184, Dow Inc.) with a weight ratio of 20:1 between the monomer to the cross-linker using a two-step molding method. A spike mold was prepared using a two-photon polymerization (2PP) three-dimensional (3D) printer (Photonic Professional GT, Nanoscribe GmbH) with a rigid commercial photo resin (IP-S, Nanoscribe GmbH). The spike mold has 9 × 7 conical spike arrays of 200 μm in height, 100 μm in diameter, 200 μm in spacing, and a 2 mm × 1.5 mm × 60 μm backing layer. We pipetted the benzophenone solution (20 wt % in ethanol, Sigma-Aldrich Inc.) over the micro-spike patch as the hydrophobic photoinitiator and coated the patch with the poly(ethylene glycol) diacrylate (PEGDA; Sigma-Aldrich Inc.) as a dissipative hydrogel layer and a chitosan-based bioadhesive as the tough adhesive for tissue surfaces sequentially. The bioadhesive for the footpads was prepared by dissolving the chitosan (high molecular weight, Sigma-Aldrich Inc.) as a bridging polymer and the unsulfated *N*-hydroxysuccinimide (98%, Sigma-Aldrich Inc.) as a coupling reagent (12 mg/ml) into the compound MES buffer (Sigma-Aldrich Inc.) at the weight ratio of 2.0 and 0.12%, respectively. The robot body was fabricated with the procedure shown in fig. S17B. First, Ecoflex 00-30 silicone rubber (mixture ratio, part A:part B = 1:1 by weight, Smooth-On Inc.) and NdFeB microparticles (average diameter, 5 μm; MQFP-15-7, Neo Magnequench) were mixed at a 1:2 ratio by weight and then poured onto apoly(-methyl methacrylate) substrate with 150-μm-thick spacers, against which a razor blade was scraped for the control of the sheet thickness. The scraped mixture was cured at 90°C on a hot plate for 30 min. The cured sheet was then cut into a 4 mm × 2 mm rectangular sheet using a laser machine (LPKF ProtoLaser U3, LPKF Laser & Electronics AG). The material has a density of 2.5 g/cm^3^ and a Young’s modulus of 163 ± 5 kPa measured by a tensile testing machine (5940 series, Instron GmbH). In fig. S17C, the footpads were bonded with the robot body with Ecoflex 00-30 silicone rubber (mixture ratio, part A:part B = 1:1 by weight, Smooth-On Inc.).

### Fabrication of the adhesive patch for sensing adhesion and pH

The adhesive patch for sensing dry adhesion had a back layer of Ecoflex-0030 silicone rubber. It was cut into a 0.5 mm × 2 mm × 100 μm rectangular sheet prepared using a laser machine (LPKF ProtoLaser U3, LPKF Laser & Electronics AG). The sheet was bonded to the middle part of the robot body using uncured Ecoflex-0030 as glue. The adhesive patch used for sensing tissue adhesion consists of three layers: the 100-μm-thick elastomer, the dissipative hydrogel, and the pH-responsive bioadhesive. The elastomer layer was prepared the same way as the used dry adhesive patch. Then, we pipetted the benzophenone solution (20 wt % in ethanol) over the patch as the hydrophobic photoinitiator and coated the patch with PEGDA as a dissipative hydrogel layer. To prepare the pH-responsive bioadhesive, 5,5’,6,6’ -tetrahydroxy-3,3,3’,3’-tetramethyl-1,1’-spirobiindane (Sigma-Aldrich Inc.), boric acid (Sigma-Aldrich Inc.), and sodium hydroxide (Sigma-Aldrich Inc.) were dissolved into deionized water with a concentration of 0.11, 0.022, and 0.02 g/ml, respectively, in the final solution. The mixture was stirred at 90°C for 8 hours for reaction and then filtrated with a 0.2-μm sterile syringe filter. Polyvinyl alcohol (molecular weight, 89,000 to 98,000, 99+% hydrolyzed; Sigma-Aldrich Inc.) and the chitosan-based bioadhesive solution were added to the solution at a weight ratio of 1:3:4 and then stirred at 90°C for 1 hour. The mixture solution (~0.2 μl) was pipetted over the hydrogel layer of the patch. The characterization result of the pH-responsive bioadhesives was shown in fig. S18.

### Fabrication of the adhesive patch for sensing tissue viscoelasticity

Benzophenone solution (20 wt % in ethanol) was pipetted over the robot body to initiate the surface bonding to hydrogels by serving as the hydrophobic photoinitiator. PEGDA was poured onto the robot body and then degassed for 30 min. After curing in a 365-nm ultra-violet chamber (ELG100S, Dinies Technologies GmbH) for 6 min, the coated robot body was washed using deionized water to remove the unreacted chemicals. The chitosan-based bioadhesive was then applied to the robot body surface by dip coating.

### Preparation of synthetic materials for adhesion measurements

The synthetic materials used for adhesion characterization included Dragon Skin 0020 (Smooth-On Inc.), Dragon Skin FxPro (Smooth-On Inc.), and Ecoflex 0030 (Smooth-On Inc.). The corresponding monomer and cross-linker were mixed at the ratio denoted as the prefix of the name of the surface and degassed for 5 min. The mixture of 2 ml was then poured into a small petri dish and cured at 60°C for 10 min. A 2-mm-thick rectangular sheet (size: 10 mm × 5 mm) was cut for adhesion tests. In addition, the 3D phantom models with surfaces of various curvatures were prepared using a 3D printer (Form 3, Formlabs Inc.) with the photo resin Clear V4 (Formlabs Inc.). Then, the Ecoflex 0030 rubber was applied to the surfaces by dip coating. The tested materials included Ecoflex 0030 with weight ratios of 1:1 and 2:1, Dragon Skin FxPro with weight ratios of 1:2 and 2:1, and Dragon Skin 0020 with a weight ratio of 1:1.

### Preparation of synthetic materials for viscoelasticity measurements

The agarose gel samples were prepared in the following steps. First, agarose powder (BioReagents A9539, Sigma-Aldrich Co.) was mixed with deionized water, after which the mixture was continuously heated and stirred at 90°C until all the powder was dissolved. Then, the solution was boiled for another 5 min, poured into a 3D-printed container (photo resin Clear V4, Formlabs Inc.) with a dimension of 25 mm × 10 mm × 10 mm (length × width × thickness), and then cooled at room temperature 24°C for about 30 min. Subsequently, the robot with the bioadhesive was attached to the gel. The same procedure was repeated for preparing agarose gel samples with varying thicknesses using different containers. The agarose gels with weight ratios of 0.3, 0.4, 0.5, 0.6, and 1.0 wt % were prepared. The gelatin powder from porcine skin (~300 g Bloom, Type A, Sigma-Aldrich Co.) was mixed with deionized water and then stirred at 90°C. After the gelatin powder was fully dissolved, the solution was cooled down at 60°C for 20 min and congealed when immersed in ice water. Thereafter, the robot was attached to the gelatin gel. The gelatin gels with weight ratios of 3.0, 4.0, and 5.0 wt % were prepared.

Sucrose was used for the preparation of viscoelastic samples (46). The sucrose powder (S9378, Sigma-Aldrich Co.) was added to the deionized water, and the mixture was heated and stirred at 90°C until all the powder was dissolved. Then, the gels were made through the same procedures above. For example, 60-0.3 agarose-based gel (weight ratio, sucrose:water:agarose, 60:40:0.3) was used for testing the material viscoelasticity in Fig. 6C. Likewise, 50-0.3, 60-0.2, 60-0.4, and 70-0.3 agarose-based gels and 50-3 and 60-3 gelatin-based gels were used. Fluorescent red polyethylene microspheres (UVPMS-BR-1.090 27-32 μm, Cospheric Co.) were adopted to visualize the deformation of the material under a fluorescent microscope (Leica M165 FC, Leica Microsystems). The mixture of the fluorescent microspheres (diameter: 27 to 32 μm) and surfactants Polysorbate 80 (P1754, Sigma-Aldrich Co.) was added to the agarose gel solution and stirred at room temperature for at least 2 min, after which the mixture was cooled for more than 30 min. The soft agarose gel sample with a rigid cylinder embedded was prepared using the following steps. First, the 1.0 wt % agarose gel solution was poured into the negative cylinder mold, whose one end was sealed by plastic tape. Then, the tape was removed, and the cured cylinder was extracted and placed on the bottom of the container. Afterward, the 0.3 wt % agarose gel solution was poured into it, and the mixture cooled at about 24°C for more than 30 min. If the cylinder needed to be at a distance from the robot, the agarose gel with a given thickness was attached to the cylinder side with the 0.3 wt % agarose gel solution. Last, the robot was attached to the desired position with the abovementioned method.

### Adhesion measurements

The adhesion measurement using the robot was conducted in the electromagnetic actuation setup, as shown in fig. S1. To make the measurement consistent for each test, a sequence of external magnetic field waveforms were designed and preprogrammed using LabVIEW (National Instruments Inc.). As shown in Fig. 4D, the procedures include three steps. First, we increased the magnetic field *B*_y_ from 0 to 10 mT rapidly to load the adhesive patch for attachment (snapshot at a time interval of 2.5 s) and maintained it for 5 s. Second, we increased the magnetic field *B*_y_ in a step-wise waveform from 0 mT up to 20 mT in the negative *y* direction with an interval of 2 mT while maintaining the magnetic field value at each step for 2.5 s to detach the adhesive patch (snapshot at a time interval of 14.2 s). Last, the measurement cycle was completed until the adhesive patch was completely detached from the tissue (snapshot at a time interval of 17.3 s). The procedures were repeated for at least five times to reduce the measurement errors.

The adhesion measurements to obtain ground-truth data were conducted in a customized experimental setup, as reported in our previous work (24). The adhesive patches, including the dry adhesive patch and the pH-responsive adhesive patch, were prepared in the same way as described in the “Fabrication of the adhesive patch for sensing adhesion and pH” section, except that the patches were cropped into 1 mm × 1 mm square sheets. They were attached to a 3D-printed tip and aligned to the substrate, as shown in fig. S3B. The tissue samples were prepared for every 10 tests (within 5 min) from the fresh animal organs to avoid the dehydration of the tissues. The approaching and retracting speeds of the probe were both set to 20 μm/s. The preload and contact time were set to 0.5 mN and 3 s, respectively.

### Viscoelasticity measurements

The soft robot attached to a tissue sample was actuated by a rotating magnetic field of different frequencies generated by the Halbach array, as shown in fig. S2. A translational motorized stage (LTS300/M, Thorlabs Inc.) mounted on the vertical stage (DIN 12897, Bochem Instrumente GmbH) was used to control the position of the Halbach array in the *x* and *z* axes, while a stepper motor (535-0372, RS Components GmbH) or DC motor (242478, Maxon Co.) was used to rotate the Halbach array.

To measure the viscoelasticity of the material, the prepared sample was first placed under the field of view of the microscope. The motorized stage moved the Halbach array to ensure that the robot was located at the center of the array. Then, the motor started rotating at a given frequency. The Halbach array was rotated at 0.1 Hz for sensing the material elasticity. Subsequently, the robot was actuated by sweeping the magnetic field from 0.3 to 15 Hz to measure the material viscoelasticity. The magnetic field magnitude was adjusted by using different Halbach arrays and changing the *z* distance from the robot to the array. The robot showed sufficient large deformation for visualization while not detaching from the material. The whole process was recorded with a high-speed camera (mmDK-2740, Dantec Dynamics A/S Inc.) at a sampling rate of 400 FPS. Five material samples were prepared for each type of material. Five soft robot samples were used for each material sample. The viscoelasticity of the samples was characterized using the oscillation frequency module on a rheometer (Discovery HR-2, TA Instruments). The constant strain was set to 0.1%. The frequency varied from 0.1 to 100 rad/s with five test points between 1 and 10 in a log scale. The gel and tissue samples were prepared as 2-mm-thick circular plates with a diameter of 20 mm. The storage and loss moduli used as the ground truth data for quantifying the sensing accuracy of our soft robot–based method are the mean values of those measured at the frequency of 0.1 Hz.

### Robot shape tracking and analysis

The robot shape tracking was achieved by extracting its edge or segmenting the image plus centerline extraction at each frame, during which the robot footpads were also tracked. A range of discrete points were sampled at the same interval from the extracted centerline or edge and further used to fit the B-spline to represent the robot curve. All the procedures were accomplished by a customized python code, which is available on GitHub (https://github.com/wchunxiang/biomechanics-estimation).

### Visualization and analysis of surface material deformation

Digital image correlation implemented with a customized python code was used to compute the displacement field of the material, whose deformation was visualized by fluorescent particles. On the basis of the assumption that there was no flip between the robot body and the tested material, the displacement of the tracked particles and the robot were combined to compute the strain field. The displacement in the *x* and *y* axes of the point $\left[x_{0}^{\left(i\right)},y_{0}^{\left(i\right)}\right]$ in the displacement field were calculated by $u_{x}\left[x_{0}^{\left(i\right)},y_{0}^{\left(i\right)},t=j\right]=x_{j}^{\left(i\right)}-x_{0}^{\left(i\right)}$ and $u_{y}\left[x_{0}^{\left(i\right)},y_{0}^{\left(i\right)},t=j\right]=y_{j}^{\left(i\right)}-y_{0}^{\left(i\right)}$, where $\left[x_{j}^{\left(i\right)},y_{j}^{\left(i\right)}\right]$ was the position of the *i*th particle or robot curve point in the *j*th frame and $\left[x_{0}^{\left(i\right)},y_{0}^{\left(i\right)}\right]$ was the original position of the tracked particle or robot curve point with no magnetic field actuation. Further, the discrete field points were processed by linear interpolation and data smoothing to obtain the displacement field, as shown in fig. S19 and movie S3. In Fig. 6H and fig. S12, the value of the maximum displacement field Δ*u*(*x, y*) at position (*x, y*) was calculated by

(1) $$\text{Δ}u(x,y)=\sqrt{{\left\{\max\left[u_{x}(x,y,t)\right]-\min\left[u_{x}(x,y,t)\right]\right\}}^{2}+{\left\{\max\left[u_{y}(x,y,t)\right]-\min\left[u_{y}(x,y,t)\right]\right\}}^{2}}$$

The wavelet transform used for the analysis of the effect of the cylinder inside the homogeneous bulk material was based on the default function cwt (Continuous 1-D wavelet transform) of the SciPy package (47).

### Correction of the robot curve misalignment for viscoelasticity sensing

As illustrated in fig. S9, the error in the extracted robot shape due to the misalignment between the imaging coordinate system *xyz* and the robot–tissue coordinate system *x’y’z’* about the *x* axis can be corrected using the following equation

(2) $$\left[\begin{array}{l} x\\ y\\ z\\ 1 \end{array}\right]=\left[\begin{array}{cccc} 1 & 0 & 0 & x_{0}\\ 0 & \cos(\varphi ) & -\sin(\varphi ) & y_{0}\\ 0 & \sin(\varphi ) & \cos(\varphi ) & z_{0}\\ 0 & 0 & 0 & 1 \end{array}\right]\left[\begin{array}{l} x^{\prime }\\ y^{\prime }\\ z^{\prime }\\ 1 \end{array}\right]$$

where (*x*_0_, *y_0_, z_0_)* is the origin coordinate of *x*’*y’z’* in *xyz* and *φ* is the misalignment angle about the *x* axis, *φ* = arcsin [(*l* – *t*)/*w*]. Here, *l* is the nominal thickness of the robot, and *t* and *w* are the real thickness and width of the robot body. Assuming that the robot only deforms in the *x’y’* plane during the actuation, the real robot displacement $u_{y}^{\prime }$ can be obtained using the correction, $u_{y}^{\prime }=u_{y}/\cos(\varphi )$, where *u_y_* is the detected robot displacement in the imaging coordinate system.

### X-ray medical imaging

An x-ray imaging device (XPERT 80, KUBTEC, Stratford CT) was used to visualize the robot shape changes on tissues. As illustrated in fig. S20A, sensing adhesion under x-ray imaging was achieved by adjusting the position and orientation of the permanent magnet. With the Halbach array mounted on a rotary motion stage in fig. S20B, the robot motion for viscoelasticity sensing was clearly demonstrated in movie S5. The x-ray accelerating voltage was set to 65 kV during the experiments, and the video was recorded at 30 FPS. The x-ray dose was measured with the radiation meter (RM-400, Voltcraft GmbH) under the α + γ + β mode for the x-ray accelerating voltage 65 kV and current 86 μA.

### Preparation of animal organs

The porcine organs in the GI tract, including the small intestine, stomach, and chicken breast, were from the local food factory in Stuttgart, Germany. The rat stomachs were from the Anatomy and Cell Biology Laboratory in University of Ulm, Germany. The organs were minimally cleaned by emptying the organ while minimizing the surface damage. The tissues used for tests and characterization were within 24 hours and stored in a refrigerator at 2°C. The organs were cut into blocks with a size of 25 mm × 15 mm (width × height) and a thickness of 6.4 ±1.4 mm. Then, the robot was attached to the surface to sense viscoelasticity. For visco-elasticity characterization, the organs were cut into circular plates with a radius of 10 mm and a thickness of 5 mm. For the adhesion tests, the organs were cut and attached to a glass slide. Then, the robot was put on the tissue to test the adhesion property.

### Preparation of mice with TNK1 expression–related intestinal disorder or disease model

The expression of TNK1 in 8-week-old *Tnk1*-knockin mice (*Rosa26rtTA/+, Hprt MycTnk1tg*) was induced by the single intraperitoneal injection of doxycycline (50 μg/g). The control group of healthy mice was treated with the intraperitoneal injection of the same volume of saline solution (0.9% NaCl). Twenty-four hours after the injection, the mice were sacrificed and dissected. Genetic modification of TNK1-knockin mice was verified by genotyping. To prepare the tissues for the viscoelasticity tests, the colon and the intestinal organs behind it were cut out, and the robot was attached to the internal surface of the opened colon. To prepare the tissues for the adhesion tests, the stomach was opened and minimally cleaned by emptying the inner stuff. All tests were completed within 3 hours.

## Supplementary Material

Supplemental Movie 1

Supplemental Movie 2

Supplemental Movie 3

Supplemental Movie 4

Supplemental Movie 5

Supplementary Materials (Notes, Figs., Legends, References)

## Data Availability

All data needed to evaluate the conclusions in the paper are present in the paper and/or the Supplementary Materials.

## References

[1] Thompson AJ, Pillai EK, Dimov IB, Foster SK, Holt CE, Franze K (2019). Rapid changes in tissue mechanics regulate cell behaviour in the developing embryonic brain. eLife.

[2] Dance A (2021). The secret forces that squeeze and pull life into shape. Nature.

[3] Läubli NF, Burri JT, Marquard J, Vogler H, Mosca G, Vertti-Quintero N, Shamsudhin N, deMello A, Grossniklaus U, Ahmed D, Nelson BJ (2021). 3D mechanical characterization of single cells and small organisms using acoustic manipulation and force microscopy. Nat Commun.

[4] Lin M, Hu H, Zhou S, Xu S (2022). Soft wearable devices for deep-tissue sensing. Nat Rev Mater.

[5] Song E, Huang Y, Huang N, Mei Y, Yu X, Rogers JA (2022). Recent advances in microsystem approaches for mechanical characterization of soft biological tissues. Microsyst Nanoeng.

[6] Kennedy KM, Chin L, McLaughlin RA, Latham B, Saunders CM, Sampson DD, Kennedy BF (2015). Quantitative micro-elastography: Imaging of tissue elasticity using compression optical coherence elastography. Sci Rep.

[7] Sigrist RMS, Liau J, El Kaffas A, Chammas MC, Willmann JK (2017). Ultrasound elastography: Review of techniques and clinical applications. Theranostics.

[8] Shung KK (2005). Diagnostic Ultrasound: Imaging and Blood Flow Measurements.

[9] Low G, Kruse SA, Lomas DJ (2016). General review of magnetic resonance elastography. World J Radiol.

[10] Schrank F, Warmuth C, Görner S, Meyer T, Tzschätzsch H, Guo J, Uca YO, Elgeti T, Braun J, Sack I (2020). Real-time MR elastography for viscoelasticity quantification in skeletal muscle during dynamic exercises. Magn Reson Med.

[11] Hu H, Zhu X, Wang C, Zhang L, Li X, Lee S, Huang Z, Chen R, Chen Z, Wang C, Gu Y (2018). Stretchable ultrasonic transducer arrays for threedimensional imaging on complex surfaces. Sci Adv.

[12] Wang C, Li X, Hu H, Zhang L, Huang Z, Lin M, Zhang Z, Yin Z, Huang B, Gong H, Bhaskaran S (2018). Monitoring of the central blood pressure waveform via a conformal ultrasonic device. Nat Biomed Eng.

[13] Wang C, Chen X, Wang L, Makihata M, Liu H-C, Zhou T, Zhao X (2022). Bioadhesive ultrasound for long-term continuous imaging of diverse organs. Science.

[14] Yu X, Wang H, Ning X, Sun R, Albadawi H, Salomao M, Silva AC, Yu Y, Tian L, Koh AC, Lee M (2018). Needle-shaped ultrathin piezoelectric microsystem for guided tissue targeting via mechanical sensing. Nat Biomed Eng.

[15] Song E, Xie Z, Bai W, Luan H, Ji B, Ning X, Xia Y, Baek JM, Lee Y, Avila R, Chen J-H-Y (2021). Miniaturized electromechanical devices for the characterization of the biomechanics of deep tissue. Nat Biomed Eng.

[16] Shin J, Yan Y, Bai W, Xue Y, Gamble P, Tian L, Kandela I, Haney CR, Spees W, Lee Y, Choi M (2019). Bioresorbable pressure sensors protected with thermally grown silicon dioxide for the monitoring of chronic diseases and healing processes. Nat Biomed Eng.

[17] Bredenoord AJ (2008). Impedance-pH monitoring: New standard for measuring gastro-oeso-phageal reflux. Neurogastroenterol Motil.

[18] Ciuti G, Caliò R, Camboni D, Neri L, Bianchi F, Arezzo A, Koulaouzidis A, Schostek S, Stoyanov D, Oddo CM, Magnani B (2016). Frontiers of robotic endoscopic capsules: A review. J Microbio Robot.

[19] Min J, Yang Y, Wu Z, Gao W (2020). Robotics in the gut. Adv Ther.

[20] Shamsudhin N, Zverev VI, Keller H, Pane S, Egolf PW, Nelson BJ, Tishin AM (2017). Magnetically guided capsule endoscopy. Med Phys.

[21] Baltsavias S, Van Treuren W, Weber MJ, Charthad J, Baker S, Sonnenburg JL, Arbabian A (2020). In vivo wireless sensors for gut microbiome redox monitoring. IEEE Trans Biomed Eng.

[22] Serwane F, Mongera A, Rowghanian P, Kealhofer DA, Lucio AA, Hockenbery ZM, Campàs O (2016). In vivo quantification of spatially varying mechanical properties in developing tissues. Nat Methods.

[23] Uslu FE, Davidson CD, Mailand E, Bouklas N, Baker BM, Sakar MS (2021). Engineered extracellular matrices with integrated wireless microactuators to study mechanobiology. Adv Mater.

[24] Wu Y, Dong X, Kim JK, Wang C, Sitti M (2022). Wireless soft millirobots for climbing threedimensional surfaces in confined spaces. Sci Adv.

[25] Blelloch ND, Yarbrough HJ, Mirica KA (2021). Stimuli-responsive temporary adhesives: Enabling debonding on demand through strategic molecular design. Chem Sci.

[26] Yan D, Pezzulla M, Cruveiller L, Abbasi A, Reis PM (2021). Magneto-active elastic shells with tunable buckling strength. Nat Commun.

[27] Li J, Celiz AD, Yang J, Yang Q, Wamala I, Whyte W, Seo BR, Vasilyev NV, Vlassak JJ, Suo Z, Mooney DJ (2017). Tough adhesives for diverse wet surfaces. Science.

[28] Huang J, Liu Y, Yang Y, Zhou Z, Mao J, Wu T, Liu J, Cai Q, Peng C, Xu Y, Zeng B (2021). Electrically programmable adhesive hydrogels for climbing robots. Sci Robot.

[29] Akram Bhuiyan MS, Roland JD, Liu B, Reaume M, Zhang Z, Kelley JD, Lee BP (2020). In situ deactivation of catechol-containing adhesive using electrochemistry. J Am Chem Soc.

[30] Machida T (1981). A study of intragastric pH in patients with peptic ulcer-with special reference to the clinical significance of basal pH value. Gastroenterol Jpn.

[31] Handorf AM, Zhou Y, Halanski MA, Li W-J (2015). Tissue stiffness dictates development, homeostasis, and disease progression. Organogenesis.

[32] Stoltz DA, Meyerholz DK, Welsh MJ (2015). Origins of cystic fibrosis lung disease. N Engl J Med.

[33] Miyahara N, Kokubo T, Hara Y, Yamada A, Koike T, Arai Y (2016). Evaluation of X-ray doses and their corresponding biological effects on experimental animals in cone-beam micro-CT scans (R-mCT2). Radiol Phys Technol.

[34] Armacki M, Trugenberger AK, Ellwanger AK, Eiseler T, Schwerdt C, Bettac L, Langgartner D, Azoitei N, Halbgebauer R, Groß R, Barth T (2018). Thirty-eight-negative kinase 1 mediates trauma-induced intestinal injury and multi-organ failure. J Clin Investig.

[35] Zhang J, Guo Y, Hu W, Soon RH, Davidson ZS, Sitti M (2021). Liquid crystal elastomer-based magnetic composite films for reconfigurable shape-morphing soft miniature machines. Adv Mater.

[36] Thitaikumar A, Krouskop TA, Ophir J (2007). Signal-to-noise ratio, contrast-to-noise ratio and their trade-offs with resolution in axial-shear strain elastography. Phys Med Biol.

[37] Delaunay R, Hu Y, Vercauteren T (2020). Medical Image Computing and Computer Assisted In-tervention-MICCAI 2020.

[38] Mirzaei M, Asif A, Rivaz H (2019). Combining total variation regularization with window-based time delay estimation in ultrasound elastography. IEEE Trans Med Imaging.

[39] Kim S-J, Lee D-S, Kim I-G, Sohn D-W, Park J-Y, Choi B-K, Kim S-W (2012). Evaluation of the biocompatibility of a coating material for an implantable bladder volume sensor. Kaohsiung J Med Sci.

[40] Chung H-J, Parsons AM, Zheng L (2021). Magnetically controlled soft robotics utilizing elastomers and gels in actuation: A review. Adv Intell Syst.

[41] Armstrong D (2004). Review article: Gastric pH—The most relevant predictor of benefit in reflux disease?. Aliment Pharmacol Ther.

[42] Ebihara T, Venkatesan N, Tananka R, Ludwig MS (2000). Changes in extracellular matrix and tissue viscoelasticity in bleomycin-induced lung fibrosis. Am J Respir Crit CareMed.

[43] Nia HT, Munn LL, Jain RK (2020). Physical traits of cancer. Science.

[44] Merki HS, Fimmel CJ, Walt RP, Harre K, Röhmel J, Witzel L (1988). Pattern of 24 hour in-tragastric acidity in active duodenal ulcer disease and in healthy controls. Gut.

[45] Narkar AR, Kendrick C, Bellur K, Leftwich T, Zhang Z, Lee BP (2019). Rapidly responsive smart adhesive-coated micropillars utilizing catechol-boronate complexation chemistry. Soft Matter.

[46] Maurer S, Junghans A, Vilgis TA (2012). Impact of xanthan gum, sucrose and fructose on the viscoelastic properties of agarose hydrogels. Food Hydrocoll.

[47] Virtanen P, Gommers R, Oliphant TE, Haberland M, Reddy T, Cournapeau D, Burovski E, Peterson P, Weckesser W, Bright J, van der Walt SJ (2020). SciPy 1.0 Contributors, SciPy 1.0: Fundamental algorithms for scientific computing in Python. Nat Methods.

[48] Nocedal J, Wright SJ (1999). Numerical Optimization.

[49] Kim Y, Yuk H, Zhao R, Chester SA, Zhao X (2018). Printing ferromagnetic domains for untethered fast-transforming soft materials. Nature.

[50] Johnson KL (1987). Contact Mechanics.

