# Supplementary material for: In situ sensing physiological properties of biological tissues using wireless miniature soft robots
Source: Sci Adv. 2023 Jun 7;9(23):eadg3988. doi: 10.1126/sciadv.adg3988 (PMC7614673; doi:10.1126/sciadv.adg3988)
Supplement: Supplementary file 1 — Notes S1 to S3 Figs. S1 to S23 Legends for movies S1 to S5 References [file sciadv.adg3988_sm.pdf]

Science Advances
AAAS

Supplementary Materials for

In situ sensing physiological properties of biological tissues using wireless miniature soft robots

Chunxiang Wang *et al.*

Corresponding author: Xiaoguang Dong, xiaoguang.dong@vanderbilt.edu; Metin Sitti, sitti@is.mpg.de

Sci. Adv. **9**, eadg3988 (2023)
DOI: 10.1126/sciadv.adg3988

The PDF file includes:

Notes S1 to S3
Figs. S1 to S23
Legends for movies S1 to S5
References

Other Supplementary Material for this manuscript includes the following:

Movies S1 to S5

Supplementary Note 1. Estimating robot-tissue adhesion.

The adhesion applied on the robot adhesive patch from the tissue surface could be estimated by the static shape of the robot body using the Euler-Bernoulli Beam theory. When the adhesive patch attaches to the tissue surface, external forces include the three-point contact forces from the tissues (**Figs. 4B**, **C**). When the adhesive patch detaches from the surface, external forces include only two-point contact forces (**Fig. 4D**). In both cases, we can use the Euler-Bernoulli Beam model to describe the mechanical behavior of the robot body. We focus on the first case as we want to estimate the adhesion applied from the tissue surface to the adhesive patch, given the static shape of the robot body and the external magnetic field. First, for the robot body, given an infinitesimal element $[s, s+\mathrm{d}s]$ (**Fig. 4B**), the moment balance equation at a static state in Eulerian space is given by,

$$M_b(s+\mathrm{d}s) - M_b(s) + \tau_m(s)A_{\text{cross}}\mathrm{d}s = \left(F_v(s) + \frac{\partial F_v}{\partial s}\mathrm{d}s\right)\mathrm{d}s\cos\theta \ - \left(F_h(s) + \frac{\partial F_h}{\partial s}\mathrm{d}s\right)\mathrm{d}s\sin\theta\,. \quad (1)$$

$\tau_m(s)$ is the magnetic torque per volume. $F_h(s)$ and $F_v(s)$ are the internal forces. $M_b(s) = EI\frac{\partial\theta}{\partial s}$ is the bending moment. $A_{\text{cross}} = wt$ is the cross area. $\theta(s)$ is the rotation angle, E is the Youngs' modulus, $I = t_b^3 w/12$ is the second moment of the cross-section area (w: width, t_b: thickness). Neglecting the high-order terms, we have

$$M_b(s+\mathrm{d}s) - M_b(s) + \tau_m(s)A_{\text{cross}}\mathrm{d}s = F_v(s)\mathrm{d}s\cos\theta - F_h(s)\mathrm{d}s\sin\theta\,. \quad (2)$$

The force balance equation is given by

$$-\frac{1}{A_{cross}}\frac{\partial F_h(s,t)}{\partial s} = 0, \quad (3)$$

$$-\frac{1}{A_{cross}}\frac{\partial F_v(s,t)}{\partial s} = \rho g\,. \quad (4)$$

If we assume the gravity of an infinitesimal element is negligible compared with the adhesion during the detachment, then we have

$$F_v(s) = F_{1y},\ s \in \left[0, \frac{L}{2} - \frac{L_0}{2}\right],\ F_v(s) = F_{2y},\ s \in \left[\frac{L}{2} + \frac{L_0}{2}, L\right], \quad (5)$$

$$F_h(s) = F_{1x},\ s \in \left[0, \frac{L}{2} - \frac{L_0}{2}\right],\ F_h(s) = F_{2x},\ s \in \left[\frac{L}{2} + \frac{L_0}{2}, L\right]. \quad (6)$$

L and L_0 are the robot length and adhesive patch length, respectively. We integrate Eqn. (2) over $s \in \left[0, \frac{L}{2} - \frac{L_0}{2}\right]$ and $s \in \left[\frac{L}{2} + \frac{L_0}{2}, L\right]$, and have Eqns. (7) and (8), respectively.

$$\int_0^{s_l} -\frac{\partial M_b(s)}{\partial s}ds = \int_0^{s_l}\tau_m(s)A_{cross}ds - \int_0^{s_l}F_v(s)\cos\theta ds + \int_0^{s_l}F_h(s)\sin\theta\, ds$$

$$\Rightarrow -M_b(s_l) + M_b(0) = \tau_{net,l}(s_l) - F_{1y}\int_0^{s_l}\cos\theta ds + F_{1x}\int_0^{s_l}\sin\theta ds$$

$$\Rightarrow M_b(0) - M_b(s_l) = \tau_{net,l}(s_l) + F_{1y}d_{1x}(s_l) - F_{1x}d_{1y}(s_l) \quad (7)$$

$d_{1x}(s_l)$, $d_{1y}(s_l)$ are the *x* and *y* components of the distance between the robot foot pad 1 and the position s_l when adhering to the tissue surface. $\tau_{net,l}(s_l) = \int_0^{s_l} \tau_m(s) A_{cross} ds$.

$$\int_{s_r}^{L} -\frac{\partial M_b(s)}{\partial s} ds = \int_{s_r}^{L} \tau_m(s) A_{cross} ds - \int_{s_r}^{L} F_v(s) \cos\theta ds + \int_{s_r}^{L} F_h(s) \sin\theta \, ds$$

$$\Rightarrow -M_b(L) + M_b(s_r) = \tau_{net,r}(s_r) - F_{2y} \int_{s_r}^{L} \cos\theta ds + F_{2x} \int_{s_r}^{L} \sin\theta \, ds$$

$$\Rightarrow M_b(s_r) - M_b(L) = \tau_{net,r}(s_r) + F_{2y} d_{2x}(s_r) - F_{2x} d_{2y}(s_r) \quad (8)$$

$d_{2x}(s_r)$, $d_{2y}(s_r)$ are the *x* and *y* components of the distance between the position s_r and the robot foot pad 2 when adhering to the tissue surface. $\tau_{net,r}(s_r) = \int_{s_r}^{L} \tau_m(s) A_{cross} ds$.

Based on the pinned-pined boundary conditions at the two ends of the robot body, we have

$$\frac{\partial \theta}{\partial s}(0) = 0, \quad (9)$$

$$\frac{\partial \theta}{\partial s}(L) = 0. \quad (10)$$

From Eqns. (7-10) we have

$$-M_b(s_l) = \tau_{net,l}(s_l) + F_{1y} d_{1x}(s_l) - F_{1x} d_{1y}(s_l), \quad (11)$$

$$M_b(s_r) = \tau_{net,r}(s_r) + F_{2y} d_{2x}(s_r) - F_{2x} d_{2y}(s_r). \quad (12)$$

According to the static shape of the robot body $\theta(s)$, $\frac{\partial \theta}{\partial s}$, $\tau_{net,l}(s_l)$, $\tau_{net,r}(s_r)$, $M_b(s_l)$, $M_b(s_r)$ could be obtained. By adjusting s_l, s_r with a set of values $\{s_{l,1}, s_{l,2} \cdots, s_{l,m}\}$ and $\{s_{r,1}, s_{r,2} \cdots, s_{r,k}\}$, multiple equations of (11) and (12) can be obtained, which can be written in the following matrix form:

$$\begin{bmatrix} -d_{1y}(s_{l,1}) & d_{1x}(s_{l,1}) \\ -d_{1y}(s_{l,2}) & d_{1x}(s_{l,2}) \\ -d_{1y}(s_{l,3}) & d_{1x}(s_{l,3}) \\ \vdots & \vdots \\ -d_{1y}(s_{l,m}) & d_{1x}(s_{l,m}) \end{bmatrix} \begin{bmatrix} F_{1x} \\ F_{1y} \end{bmatrix} = \begin{bmatrix} -M_b(s_{l,1}) - \tau_{net,l}(s_{l,1}) \\ -M_b(s_{l,2}) - \tau_{net,l}(s_{l,2}) \\ -M_b(s_{l,3}) - \tau_{net,l}(s_{l,3}) \\ \vdots \\ -M_b(s_{l,m}) - \tau_{net,l}(s_{l,m}) \end{bmatrix}, \quad (13)$$

$$\begin{bmatrix} -d_{2y}(s_{r,1}) & d_{2x}(s_{r,1}) \\ -d_{2y}(s_{r,2}) & d_{2x}(s_{r,2}) \\ -d_{2y}(s_{r,3}) & d_{2x}(s_{r,3}) \\ \vdots & \vdots \\ -d_{2y}(s_{r,k}) & d_{2x}(s_{r,k}) \end{bmatrix} \begin{bmatrix} F_{2x} \\ F_{2y} \end{bmatrix} = \begin{bmatrix} M_b(s_{r,1}) - \tau_{net,r}(s_{r,1}) \\ M_b(s_{r,2}) - \tau_{net,r}(s_{r,2}) \\ M_b(s_{r,3}) - \tau_{net,r}(s_{r,3}) \\ \vdots \\ M_b(s_{r,k}) - \tau_{net,r}(s_{r,k}) \end{bmatrix}, \quad (14)$$

where the subscript (m) and (k) represent the m-th value of s_l and k-th value of s_r.

Eqns. (13) and (14) can be represented as

$$\mathbf{D_1 f_1} = \mathbf{M_1}\,, \tag{15}$$

$$\mathbf{D_2 f_2} = \mathbf{M_2}\,. \tag{16}$$

Here, $\mathbf{D_1}, \mathbf{f_1}, \mathbf{M_1}, \mathbf{D_2}, \mathbf{f_2}, \mathbf{M_2}$ represent the corresponding matrix and vector in (13) and (14). Subsequently, $\mathbf{f_1}$ and $\mathbf{f_2}$ can be solved by employing the following optimization process:

$$\text{minimize} \quad (\mathbf{D_1 f_1} - \mathbf{M_1})^{\mathbf{T}} \mathbf{Q_1} (\mathbf{D_1 f_1} - \mathbf{M_1}), \tag{17}$$

$$\text{minimize} \quad (\mathbf{D_2 f_2} - \mathbf{M_2})^{\mathbf{T}} \mathbf{Q_2} (\mathbf{D_2 f_2} - \mathbf{M_2}), \tag{18}$$

where $\mathbf{Q_1}$ and $\mathbf{Q_2}$ are the matrices that give higher weightings to the robot body positions considered more important. The optimization process can be solved with solvers, such as quadratic optimization (*48*), which can minimize the effect of the detection noise, as shown in **fig. S21**. Furthermore, we assume the reaction torque at the patch-substrate interface is negligible, as shown in **fig. S22**. For the whole robot body, we have the force balancing equations

$$F_{1x} + F_{2x} + F_{ax} = 0, \tag{19}$$

$$F_{1y} + F_{2y} + F_{ay} = F_g, \tag{20}$$

where F_g is the gravity of the bio-adhesive patch. Therefore, with Eqns. (17-20), we could estimate F_{ax} and F_{ay}.

Supplementary Note 2. Estimating tissue viscoelastic properties.

We use the following viscoelastic constitutive equation to model the soft tissues.

$$\boldsymbol{\sigma}^{\boldsymbol{t}} = \mu_t J_t^{-\frac{5}{3}} \left(\mathbf{F_t F_t^T} - \frac{I_1}{3}\mathbf{I} \right) + K_t(J_t - 1)\mathbf{I}, \quad \Omega_t \times T \tag{21}$$

where $\boldsymbol{\sigma}^{\boldsymbol{t}}$ is the Cauchy stress of the soft material, μ_t and K_t are the material's shear modulus and bulk modulus, respectively. $\mathbf{F_t}$ represents the deformation gradient tensor. $I_1 = tr(\mathbf{FF^T})$ is the first invariant of the left Cauchy–Green tensor. The quantity J_t is the volumetric Jacobian of the deformation, as defined as $J_t = \det \mathbf{F_t}$. Ω_t is the domain of the soft tissue. T is the domain of time. For an incompressible solid, the deformation satisfies $J_t = 1$. We have the stress and strain relationship given by

$$\boldsymbol{\sigma}^{\boldsymbol{t}} = \mathbf{C_t}\boldsymbol{\epsilon}^{\mathbf{t}} + \eta_t \frac{d\epsilon^{\mathrm{t}}}{dt}, \quad \Omega_t \times T \tag{22}$$

where $\boldsymbol{\epsilon}^{\mathbf{t}}$ is the strain of the soft materials, $\mathbf{C_t}$ is the elasticity tensor, and η_t is the viscosity of the soft material. The normal stress-strain relationship of the soft material is given by

$$\sigma_{yy}^t = E_t \epsilon_{yy}^t + \eta_t \frac{d\epsilon_{yy}^t}{dt}. \tag{23}$$

We propose a frequency-sweeping method to estimate E_t and η_t. By representing the system states in the frequency domain, we have

$$\sigma_{yy}^t(j\omega) = (E_t + \eta_t j\omega)\epsilon_{yy}^t(j\omega), \tag{24}$$

$$\frac{\epsilon_{yy}^t(j\omega)}{\sigma_{yy}^t(j\omega)} = \frac{1}{j\eta_t\omega + E_t} = \frac{1}{E_t} \cdot \frac{1}{j\frac{\eta_t}{E_t}\omega + 1}, \tag{25}$$

where ω is the frequency and j is the imaginary unit. Similarly, the soft robot could also be modeled following the generalized neo Hookean model for magnetic composite materials (*49*), given by

$$\boldsymbol{\sigma}^{\boldsymbol{r}} = \mu_r J_r^{-\frac{5}{3}} \left(\mathbf{F_r F_r^T} - \frac{I_{1r}}{3}\mathbf{I} \right) + K_r(J_r - 1)\mathbf{I} - \frac{1}{J}\mathbf{B} \otimes \mathbf{F_r M}, \quad \Omega_r \times T, \tag{26}$$

$$\boldsymbol{\sigma}^{\boldsymbol{r}} = \mathbf{C^r}\boldsymbol{\epsilon}^{\mathbf{r}}, \quad \Omega_r \times T \tag{27}$$

where $\boldsymbol{\sigma}^{\boldsymbol{r}}$ is the Cauchy stress of the robot, where $\boldsymbol{\sigma}^{\boldsymbol{r}}$ is the Cauchy stress of the robot soft body, μ_r and K_r are the material's shear modulus and bulk modulus, respectively. $\mathbf{F_t}$ represents the deformation gradient tensor. $I_{1r} = tr(\mathbf{F_r F_r^T})$ is the first invariant of the left Cauchy–Green tensor. The quantity J_r is the volumetric Jacobian of the deformation, as defined as $J_r = \det \mathbf{F_r}$. Ω_r is the domain of the soft tissue. We further have the boundary conditions given by

$$\boldsymbol{\sigma}^{\boldsymbol{r}}\boldsymbol{n} = -\boldsymbol{\sigma}^{\boldsymbol{t}}\boldsymbol{n}, \quad \partial\Omega_{rt} \times T, \tag{28}$$

where $\boldsymbol{n}$ is the tissue surface normal unit vector. Given a specific robot, we assume that the normal stress of the soft tissue is proportional to the external magnetic field with a scaling factor, which could be calibrated. k_y is a function of the bulk modulus K_r and the magnetization profile $M(s)$ of the robot and independent from the external magnetic field and tissues. ϵ_{yy}^t can be estimated from the displacement of the tissue at the boundary, $u_y^t = u_y^r$,

where u_y^r and u_y^t are the robot body displacement and tissue displacement. Based on the Hertzian contact theory (*50*), the relation between the strain field distribution ϵ_{yy}^t and the robot displacement u_y^r can be assumed as the line contact on a half-plane,

$$\epsilon_{yy}^t = -\frac{2y^3}{\pi E_t}\int_a^b \frac{cE_t u_y^r(x')dx'}{[(x-x')^2+y^2]^2} = -\frac{2cy^3}{\pi}\int_a^b \frac{u_y^r(x')dx'}{[(x-x')^2+y^2]^2}, \tag{29}$$

where c is the constant that can be determined by regression, as shown in **fig. S19**.

Now we have

$$\frac{\epsilon_{yy}^t(j\omega)}{B_m(j\omega)} = \frac{k_y}{j\eta_t\omega+E_t} = \frac{k_y}{E_t}\cdot\frac{1}{j\frac{\eta_t}{E_t}\omega+1}. \tag{30}$$

Notably, the elasticity E_t and the viscosity η_t in the viscoelastic model represent the same physical terms as the storage modulus E' and the loss modulus E'' as measured by a rheometer. Considering this, we correlate the elasticity E_t with the storage modulus E' as $E_t = k_e E'$. Equation 30 is rewritten as

$$\frac{\epsilon_{yy}^t(j\omega)}{B_m(j\omega)} = \frac{k_y}{k_e E'}\cdot\frac{1}{j\frac{\eta_t}{E_t}\omega+1} = \frac{k}{E'}\cdot\frac{1}{j\frac{\eta_t}{E_t}\omega+1}. \tag{31}$$

Here, we define $k = \frac{k_y}{k_e}$, which was calibrated by the regression shown in **Fig. 6D**. We correlate the time constant $\tau = \frac{\eta_t}{E_t}$ with the ratio $\frac{E''}{E'}$ as $\tau = \frac{\eta_t}{E_t} = k_\tau\frac{E''}{E'}$. Equation (31) can be expressed as

$$\frac{\epsilon_{yy}^t(j\omega)}{B_m(j\omega)} = \frac{k}{E'}\cdot\frac{1}{j\frac{\eta_t}{E_t}\omega+1} = \frac{k}{E'}\cdot\frac{1}{j\left(\frac{k_\tau E''}{E'}\right)\omega+1} \tag{32}$$

where the coefficient k_τ was calibrated by regression, as illustrated in **Fig. 6E**.

Supplementary Note 3. Calculating the signal-to-noise ratio.

The signal for estimating the material stiffness is based on the material strain, so the signal-to-noise ratio (SNR) is defined as

$$SNR = \frac{\bar{s}}{\sigma}, \tag{33}$$

where $\bar{s}$ denotes the average of the strain values, while σ represents the standard deviation of the strain values. For ultrasound elastography, $\bar{s}$ and σ are the average and standard deviation of the strain within the sampling window, respectively (*38*).

The SNR of our proposed method was calculated with the following steps. First, the robot attached to the porcine stomach (**fig. S23A**) was placed under the X-ray imaging device (XPERT 80, KUBTEC, Stratford CT) with no magnetic field applied (**fig. S23B (i)**), after which the robot body shape was extracted using the image processing algorithm in "**Robot shape tracking and analysis**" Section in **Materials and Methods**, as shown in **fig. S23B (ii)**. Then, the tissue was deformed by the robot with a static magnetic field applied (**fig. S23C (i)**), and the extracted robot body shapes in **fig. S23B (ii)** and **fig. S23C (ii)** were used to estimate the tissue strain $\overline{\epsilon_{yy}}$ with Eqn. (29) and $\overline{\epsilon_{yy}} = \int_{0.3L}^{0.7L} \epsilon_{yy}(s) ds \,/ 0.4L$ (L is the robot length). Small variations of the extracted body shapes were observed due to the imaging noise, as shown in **fig. S23C (ii)**, which caused the fluctuation of the estimated tissue strain in **fig. 23D**. The average strain $\bar{s}$ was calculated by

$$\bar{s} = \frac{\sum_{i=1}^{N} \overline{\epsilon_{yy}}(t = t_i)}{N}, \tag{34}$$

where $\overline{\epsilon_{yy}}(t = t_i)$ is the estimated tissue strain at time t_i, and N is number of frames in the captured video. Further, the standard deviation was calculated with

$$\bar{s} = \sqrt{\frac{\sum_{i=1}^{N} \left(\overline{\epsilon_{yy}}(t = t_i) - \bar{s}\right)^2}{N}}. \tag{35}$$

The calculated SNR of our method was 39.827, with $\bar{s} = 0.162$ and $\sigma = 0.004$.

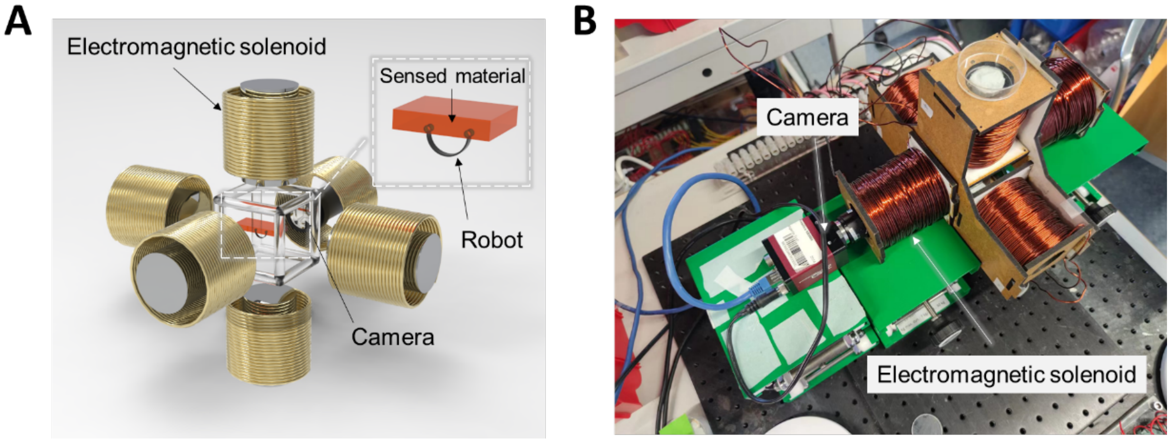

Fig. S1. The electromagnetic actuation system for controlling the soft robot. A. The schematics of the three pairs of solenoids of the actuation setup. The robot can be operated in a cubic space of a three-dimensional uniform magnetic field and a magnitude up to 30 mT. The homogeneity of the magnetic field is 93% within a 10 mm-by-10 mm-by-10 mm space, which is calibrated by the 3D magnetic field sensor (TLV493D-A1B6, Infineon GmbH). **B.** Image of the practical magnetic actuation system.

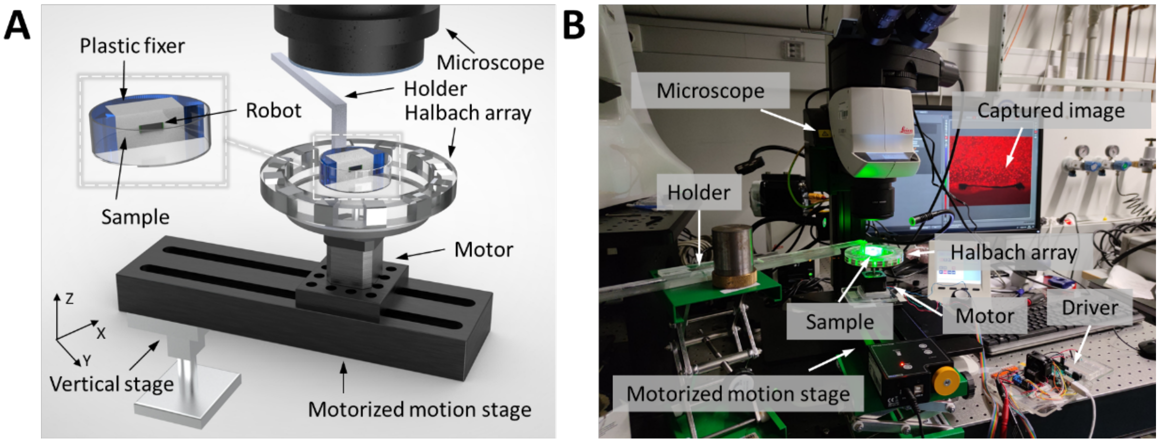

Fig. S2. Experimental setup for sensing viscoelasticity of bulk materials using wireless soft robots. **A.** The schematic of the actuation system. The Halbach array made of magnet cubes can generate a uniform magnetic field, which is connected to the stepper motor (535-0372, RS Components GmbH) or the DC motor (242478, Maxon Co.), so that a rotating uniform magnetic field can be produced. The motor is mounted on a linear translational motorized stage (LTS300/M, Thorlabs Inc.) coupled to the vertical stage (DIN 12897, Bochem Inc.) for the precise position of the Halbach array in the *x-z* plane. Four Halbach arrays made of NdFeB N42 grade magnet cubes (W-05-N, W-07-N and W-10-N, Webcraft GmbH) were prepared with a magnitude of 5.8 mT, 12.3mT, 20.1 mT and 24.2 mT in the *x-y* plane at the center. The homogeneity of the magnetic field for all Halbach arrays is over 90% within a 10 mm-by-10 mm middle region in the *x-y* plane, which is calibrated by the 3D magnetic field sensor (TLV493D-A1B6, Infineon GmbH). A fluorescent microscope (Leica M165 FC, Leica Microsystems) with the fluorescence camera (Leica DFC7000T, Leica Microsystems) or a high-speed camera (DK-2740 Skovlunde, Dantec Dynamics A/S Inc.) is placed above the Halbach array for the visualization of the robot deformation. **B.** Image of the practical actuation and imaging system.

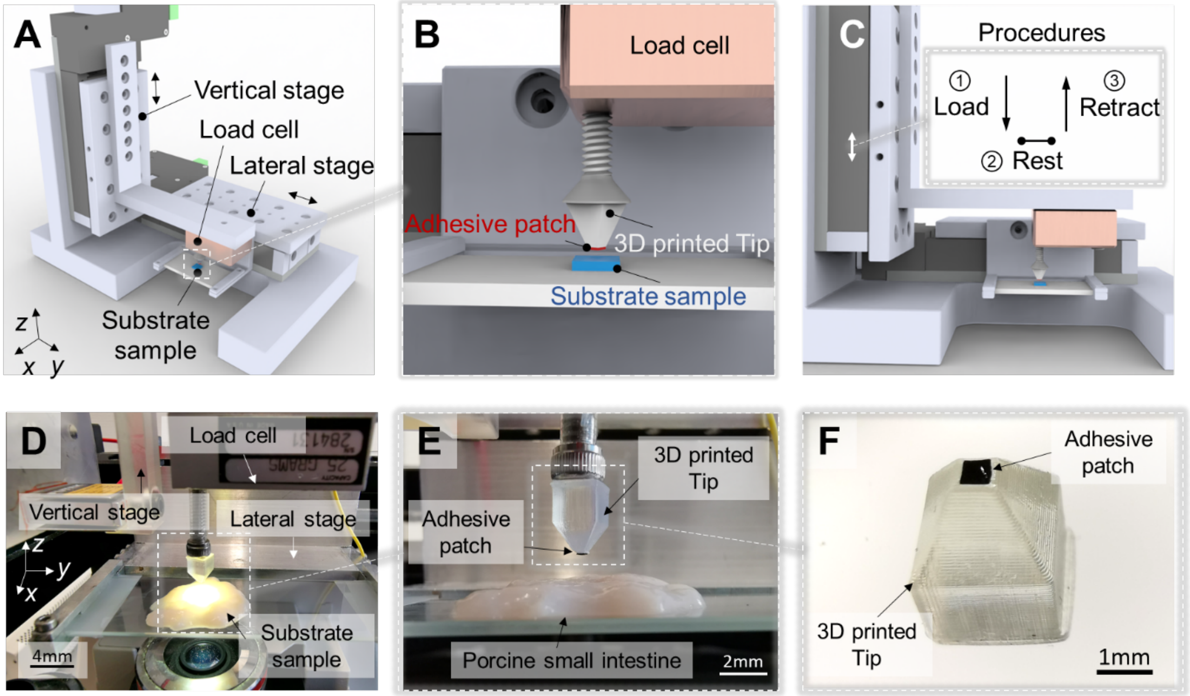

Fig. S3. Customized experimental setup for the adhesion measurement. **A.** Schematics of the setup. The adhesion was measured using a high-precision load cell (GSO-25, Transducer Techniques, LLC) mounted on a motorized vertical stage. **B.** Schematics of the sample configuration for adhesion measurement. The adhesive patch was bonded to the 3D printed probe connected to the load cell, and the substrate sample was placed on a microscope slide mounted on the lateral stage. **C.** Procedures of the adhesion measurement, including load, rest, and retract. The whole process is shown in **movie S2**. **D.** Image of the practical adhesion measurement system. Scale bar, 4 mm. **E.** Sample configuration for adhesion measurement. Substrate sample material: porcine small intestine. Scale bar, 2 mm. **F.** Adhesive patch with the pH-responsive bio-adhesive attached to a 3D printed tip (photo resin Clear V4, Formlabs, Inc.). Scale bar, 1 mm.

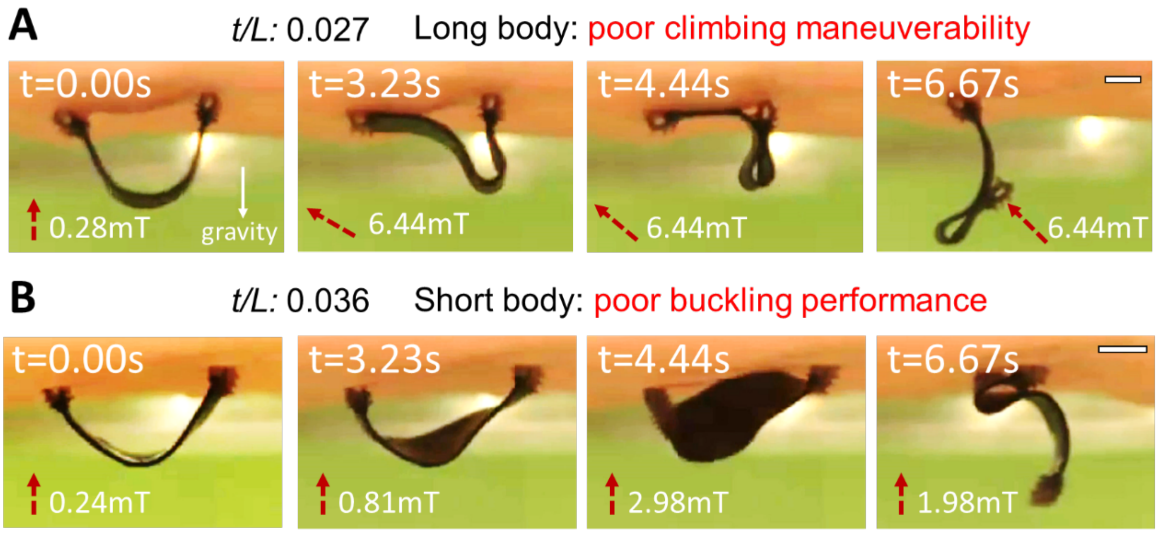

Fig. S4. Effect of the thickness-to-length ratio of the robot body design on the robot locomotion and sensing performance. A. Sequential images of a robot with a thickness-to-length ratio of 0.027 failed in climbing on porcine small intestine ex vivo. It is hard to achieve climbing locomotion for this design. **B.** Sequential images of a robot with a thickness-to-length ratio of 0.036 failed in performing adhesion sensing via buckling. In all figures, scale bars represent 1 mm.

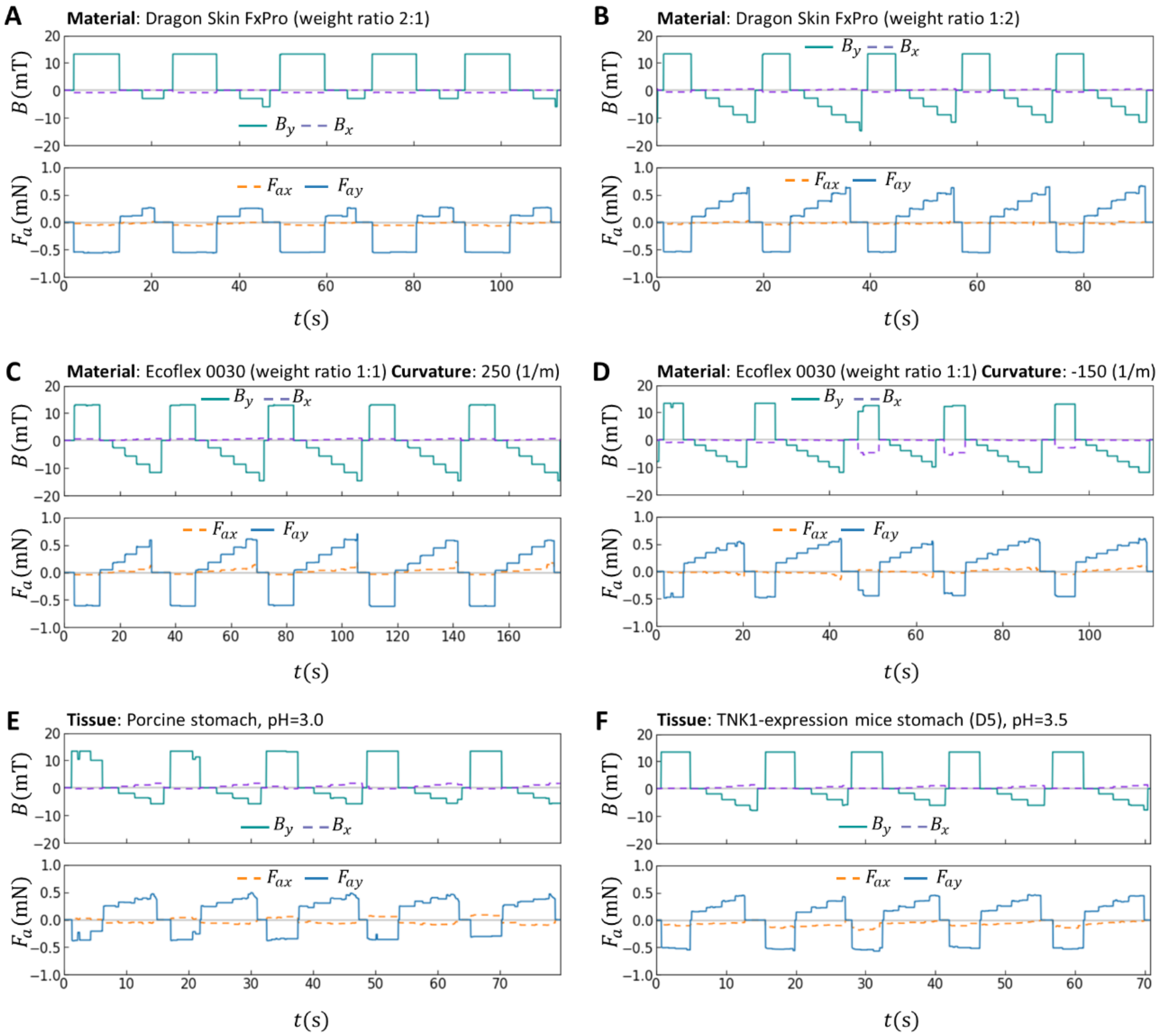

Fig. S5. The estimated force at the adhesive patch and the corresponding external magnetic field of the robot adhesion sensing over 5 repetitions. The substrate materials include **A.** Flat FxPro 2:1 surface (Dragon Skin FX Pro/1 Silicone Rubber, mixture ratio Part A: Part B = 2:1 by weight, Smooth-On Inc.), **B.** Flat FxPro 2:1 surface (Dragon Skin FX Pro/1 Silicone Rubber, mixture ratio Part A: Part B = 1:2 by weight, Smooth-On Inc.). **C.** Curved Ecoflex 0030 surface with a curvature of 250 m^{-1} (Ecoflex 0030 Silicone Rubber, mixture ratio Part A: Part B = 1:2 by weight, Smooth-On Inc.), **D.** Curved Ecoflex 0030 surface with a curvature of -150 m^{-1} (Ecoflex 0030 Silicone Rubber, mixture ratio Part A: Part B = 1:2 by weight, Smooth-On Inc.). **E.** The porcine stomach ex vivo with a pH value of 3.0, and **F.** The TNK1-expression mouse stomach ex vivo with a pH value of 3.5 (mouse number D5). The proposed method yields consistent adhesion estimation results for over 5 repetitions with an average relative standard deviation of 4.4%.

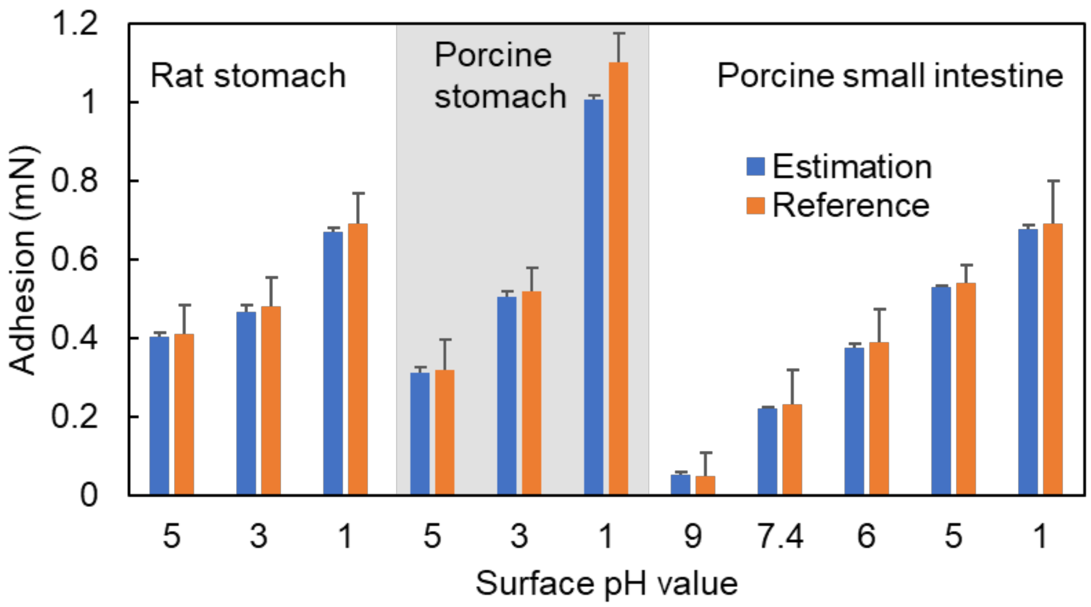

Fig. S6. The estimated robot-tissue adhesion on different tissue surfaces of various pH values. The standard phosphate-buffered saline (PBS) buffer solution of different pH values was added onto the same tissue surfaces to achieve the prescribed pH value. The error bar represents the standard deviation for $n = 5$ measurements, where n represents the number of samples.

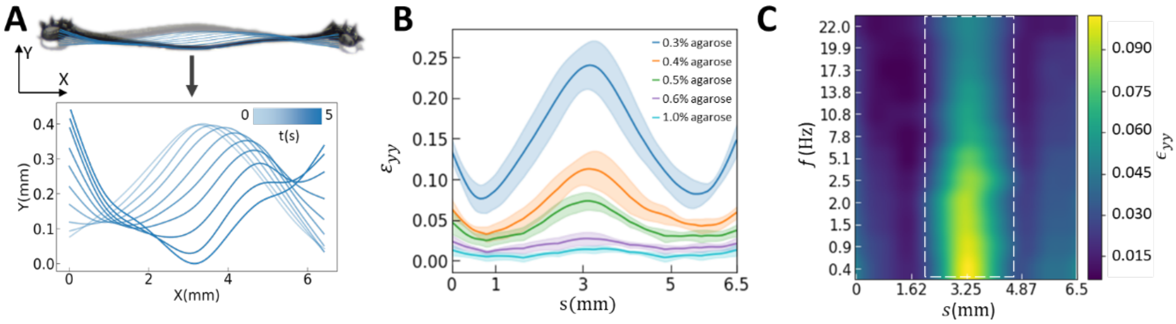

Fig. S7. Robot deformation under dynamic actuation for sensing viscoelasticity. A. Robot curves of eight frames within a half cycle under the rotating magnetic field actuation (24 mT, 0.1 Hz). Material: 0.3 wt% agarose gel. **B.** The maximum strain distribution along the robot body on different agarose gels, actuated by the rotating magnetic field (20 mT, 0.1 Hz). **C.** The maximum strain distribution along the robot body under the rotating magnetic field (26mT) of varying frequencies. The robot body, from $s = 0.3L$ to $s = 0.7L$ (robot body length $L =$ 6.5 mm), as highlighted in the white dashed rectangle, has the largest ϵ_{yy} so that the data has the largest signal-to-noise ratio. The types of noise include the noise and resolution of the image, and the detection noise of the image processing algorithm. Material: porcine fungus tissue.

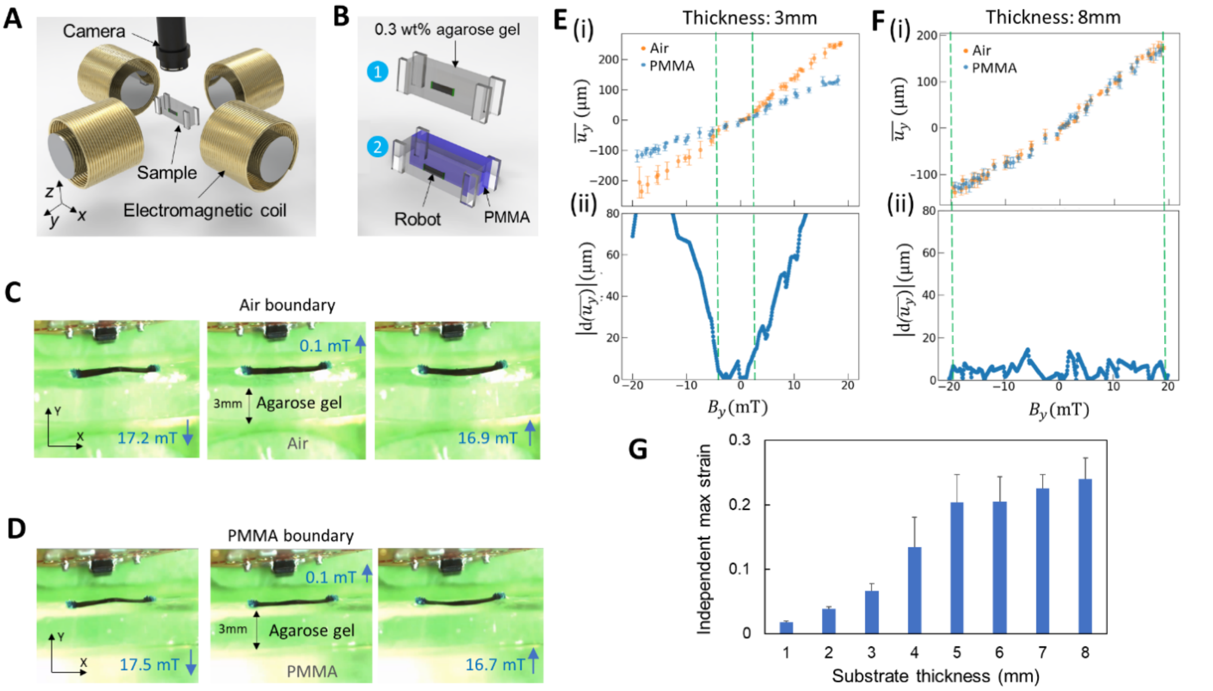

Fig. S8. Effect of the substrate material thickness on the viscoelasticity sensing. **A.** The schematics of the experimental setup used for the characterization. **B.** The schematics of the samples. The 0.3 wt% agarose gels with the size of 25 mm × 10 mm (length × width) and the thickness varying from 1 mm to 8 mm at 1 mm interval were fixed in two different ways for the 1) free and 2) fixed boundary conditions at the rear face of the substrate material. The robot was attached to the front face. **C.** The robot deformation (sample thickness: 3 mm) in 1) free boundary condition under different magnetic fields. **D.** The robot deformation (sample thickness: 3 mm) in 2) fixed boundary condition under different magnetic fields. **E.** The comparison **(i)** and the difference **(ii)** of the robot displacement $\overline{u_y}$, calculated by $\overline{u_y} = \int_{0.3L}^{0.7L} u_y(s)ds\,/0.4L$, between the two different boundary conditions under the magnetic actuation ranging from -20 mT to 20 mT for the sample in **C** and **D**. The two plots overlapped within the two green dashed lines as shown in the subplot **(i)** and the absolute difference, $|d(\overline{u_y})|$, was within the noise level of 12 μm, as shown in the subplot **(ii)**. In this case, the maximum deformation independent of the boundary condition at the thickness of 3 mm is from -19 to 25 μm, as indicated by the intersections between the green dashed lines and the curves. **F.** The comparison **(i)** and the difference **(ii)** of the robot displacement $\overline{u_y}$ for the 0.3 wt% agarose gel substrate with a thickness up to 8 mm. Actuated by the magnetic field of 20 mT, indicated by the green dashed lines, the robot reached the maximum deformation, while the absolute differences $|d(\overline{u_y})|$ were within the noise level of 12 μm. **G.** The independent maximum strain (calculated by $\overline{\epsilon_{yy}} = \int_{0.3L}^{0.7L} \epsilon_{yy}(s)ds\,/0.4L$ with the magnetic field indicated by the green lines in **E**) for 0.3 wt% agarose gel substrate as a function of the thickness. Within the independent maximum strain, the boundary conditions have minimal influence on the robot deformation under the given material thickness.

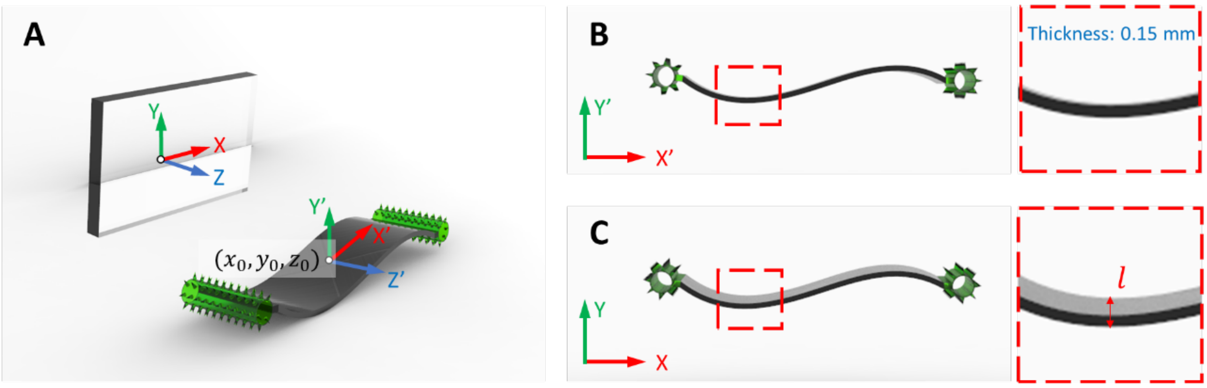

Fig. S9. Correction of the misalignment between the coordinate systems of the imaging plane and the robot. **A.** The definition of the coordinate system of the imaging plane and the robot, which are denoted as xyz and $x'y'z'$, respectively. **B.** The robot shape seen from the z' direction, where the distance between the upper and lower robot body edge equals the body thickness (0.15 mm). **C.** The robot shape seen from the z direction, where the additional thickness l can be used to characterize the rotation angle for calculating the robot body displacement in the robot coordinate system ("**Correction of the robot curve misalignment for viscoelasticity sensing**" Section in **Materials and Methods**).

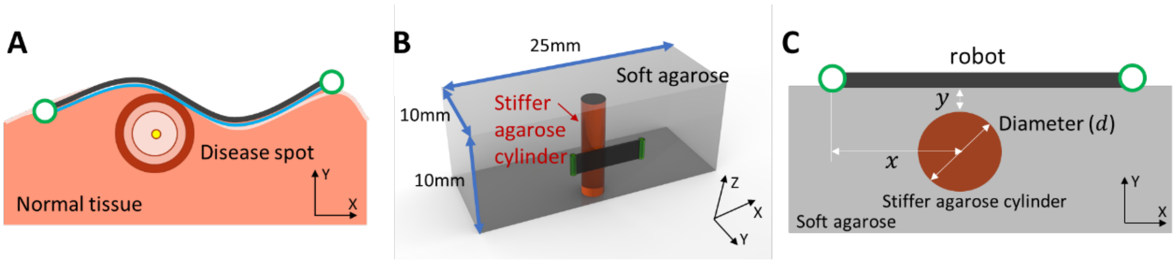

Fig. S10. Schematics of the robot sensing viscoelasticity of the non-homogeneous materials. **A.** The ability to sense the viscoelasticity of the non-homogeneous materials for the robot can be used to detect the diseased spot. **B.** A stiff cylinder made of 1.0 wt% agarose gel of various diameters is inserted into the soft bulk material made of 0.3 wt% agarose gel at different positions relative to the robot, resembling the stiffer diseased spot within the softer healthy tissues, such as the cystic fibrosis in lungs [23]. **C.** The distances between the cylinder and the robot in x and y directions, x and y, and the diameter of the cylinder, d, define the location and size of the disease spot.

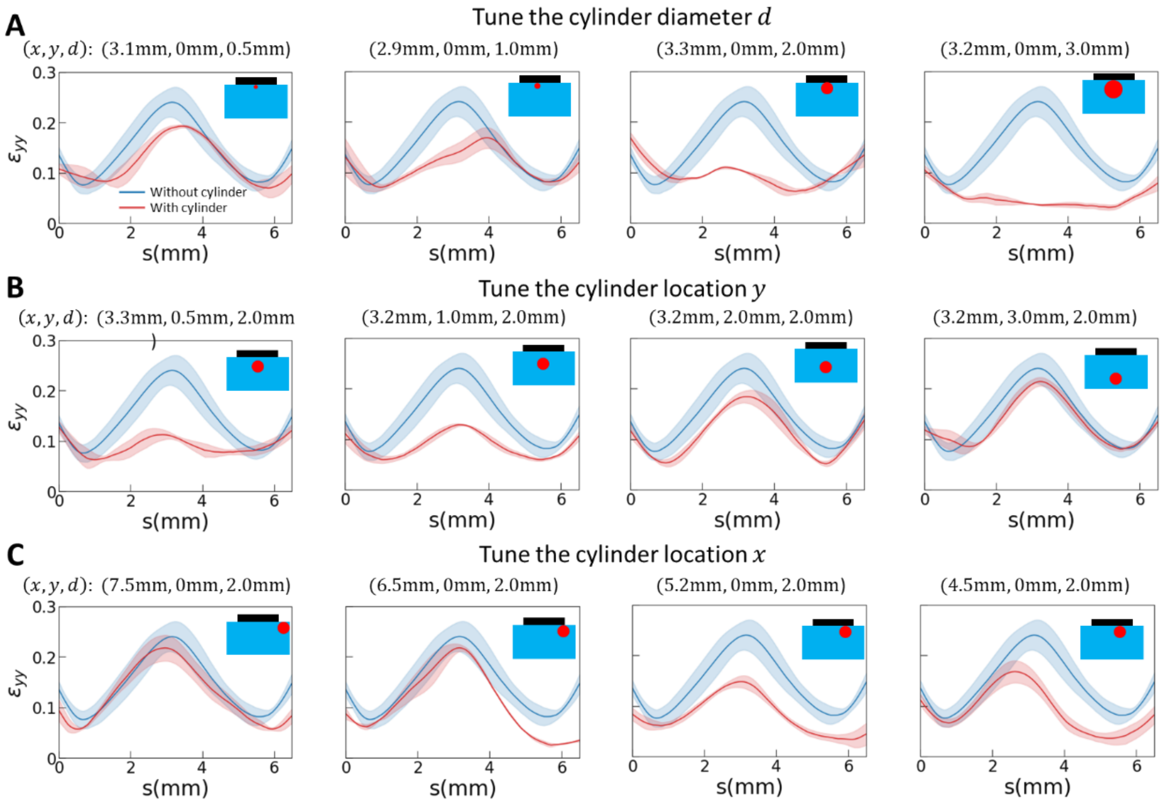

Fig. S11. Robot deformation in the presence of the stiff cylinder of various sizes at different locations under dynamic actuation for viscoelasticity sensing. A. The effect of the cylinder diameter d on the robot deformation. The schematic in each subplot indicates the location of the cylinder, where the red circle, the blue rectangle and the black line denote the stiff cylinder, the soft gel and the robot. The blue line represents the robot deformation distribution without the stiff cylinder, while the red curve plots the robot deformation distribution in the presence of the cylinder, as shown in the schematic in each subplot. **B.** The effect of the cylinder location relative to the robot in the y axis. **C.** The effect of the cylinder location relative to the robot in the x axis. In all figures, the error bar represents the standard deviation for n = 5 measurements for the curve without the cylinder and n = 3 measurements for the curves with the cylinder, where n represents the number of samples.

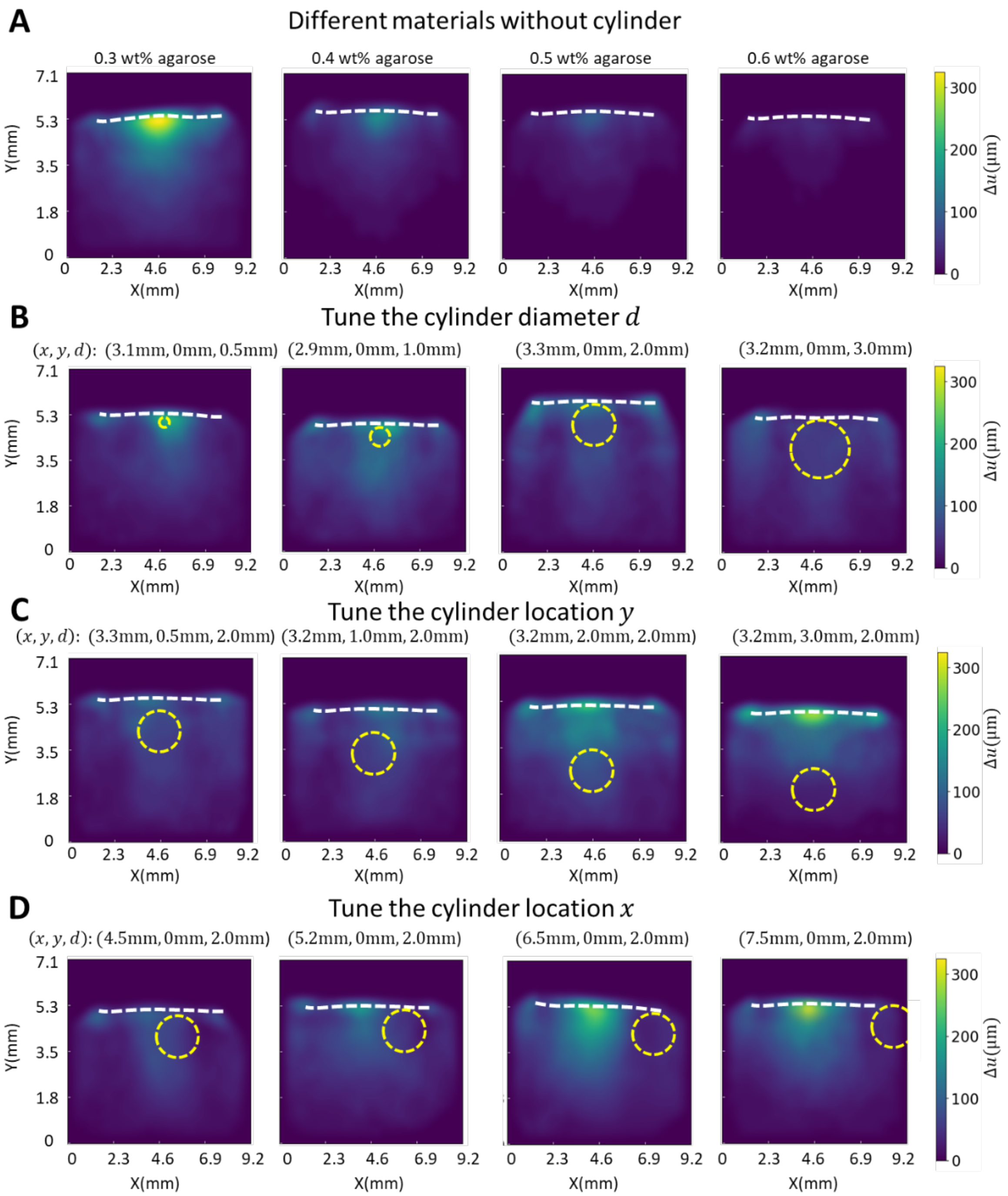

Fig. S12. Material deformation distribution in the presence of the stiff cylinder of various sizes at different locations during viscoelasticity sensing. A. Comparison of the measured maximum displacement ("**Visualization and analysis of material deformation**" Section in **Materials and Methods**) distribution for materials of different stiffness without the stiff cylinder. The white dashed line represented the robot. **B.** The effect of the cylinder diameter d on the material deformation. The yellow dashed circle indicates the cylinder. **C.** The effect of the cylinder distance to the robot in the y axis. **D.** The influence of the cylinder location relative to the robot in the x axis. The material displacement range is characterized using the customized digital imaging correlation analysis of the fluorescent (UVPMS-BR-1.090 27-32µm, Cospheric Co.) dispersed in the 0.3%wt agarose gel under the fluorescence microscope.

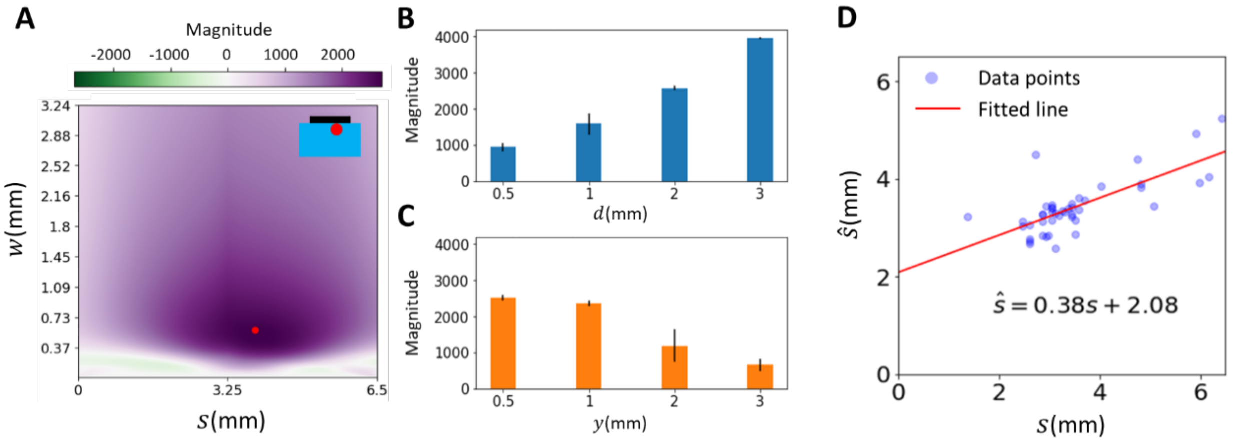

Fig. S13. Estimation of the cylinder location in the x axis and quantification of its influence on the robot deformation with the wavelet analysis during viscoelasticity sensing. A. The wavelet analysis of the difference between the robot deformation u_y in the presence of a stiff cylinder and the reference—the robot deformation u_y in the presence of no stiff cylinders (see "**Wavelet analysis of the cylinder influence**" Section in **Materials and Methods**). w denotes the wavelet base function width, while s indicates the location along the robot body. The wavelet with a large w processes a larger robot body range around s. The magnitude indicates the difference between the robot deformation with and without the cylinder, which quantifies the cylinder's influence on its surrounding area. The magnitude value is influenced by multiple factors, such as the cylinder stiffness, diameter, and distance to the robot body. The red point represents the corresponding location of the maximum magnitude. The schematic in the subplot indicates the location of the cylinder, where the red circle, the blue rectangle and the black line denote the stiff cylinder, the soft gel, and the robot. **B.** The maximum magnitudes of the wavelet analysis result of the cylinder diameter at the location $(x, y) = (3.2 \text{ mm}, 0.0 \text{ mm})$. The magnitude variable quantifies the cylinder's influence on the robot deformation. **C.** The maximum magnitudes of the wavelet analysis result of the cylinder with $d = 2.0$ mm and $y = 0.0$ mm. **D.** The correlation between the estimated cylinder location $\hat{s}$ and the practical location s. The estimation error is within 1 mm.

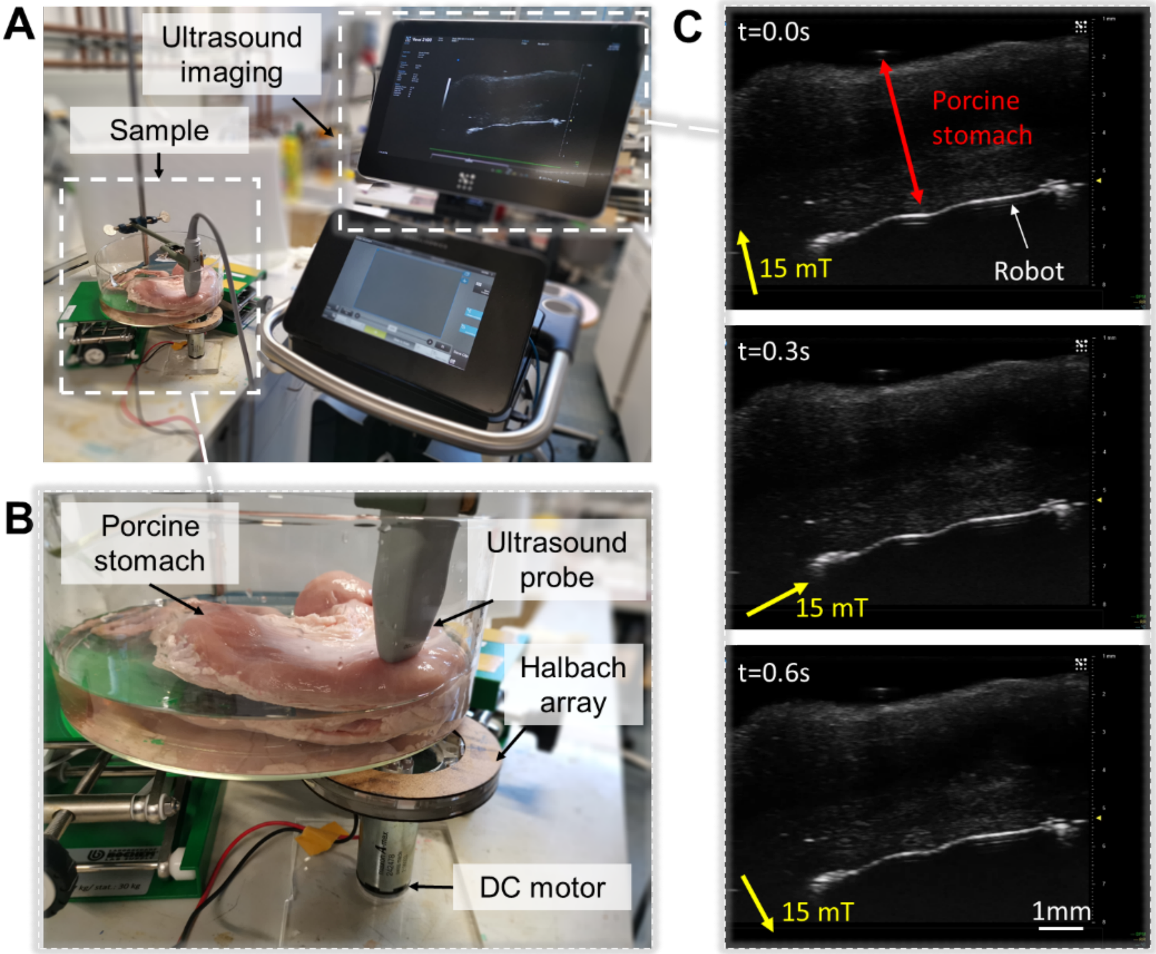

Fig. S14. Sensing tissue viscoelastic properties by robot dynamic shape under ultrasound imaging. **A.** The practical experimental setup. The robot was delivered into the porcine stomach and attached to the inner wall. The viscoelasticity sensing process was actuated by a Halbach array as shown in **fig. S2** and the robot deformation was tracked using the ultrasound imaging machine (Vevo 2100, FUJIFILM VisualSonics, Inc.). **B.** The zoomed-in image of the robot sensing the viscoelasticity of porcine stomach under the rotating magnetic field actuation. The ultrasound probe was in direct contact with the part of porcine stomach where the robot was located beneath and the Hallbach array was placed right below the robot. **C.** Video snapshots (**movie S5**) of a soft robot on the porcine stomach surface for sensing viscoelastic properties visualized by ultrasound imaging. Scale bar, 1mm.

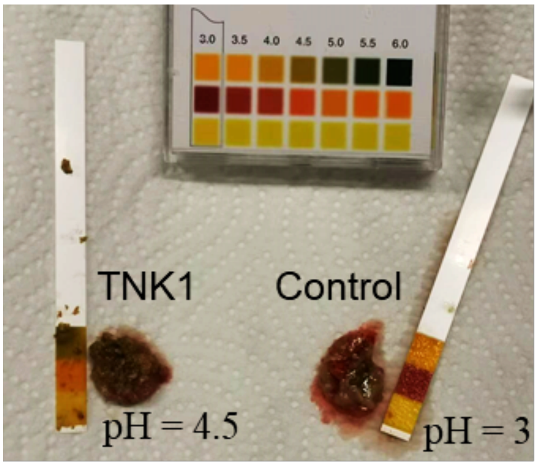

Fig. S15. The basal pH in the stomach of the TNK1-expression mouse and the mouse from the healthy control group.

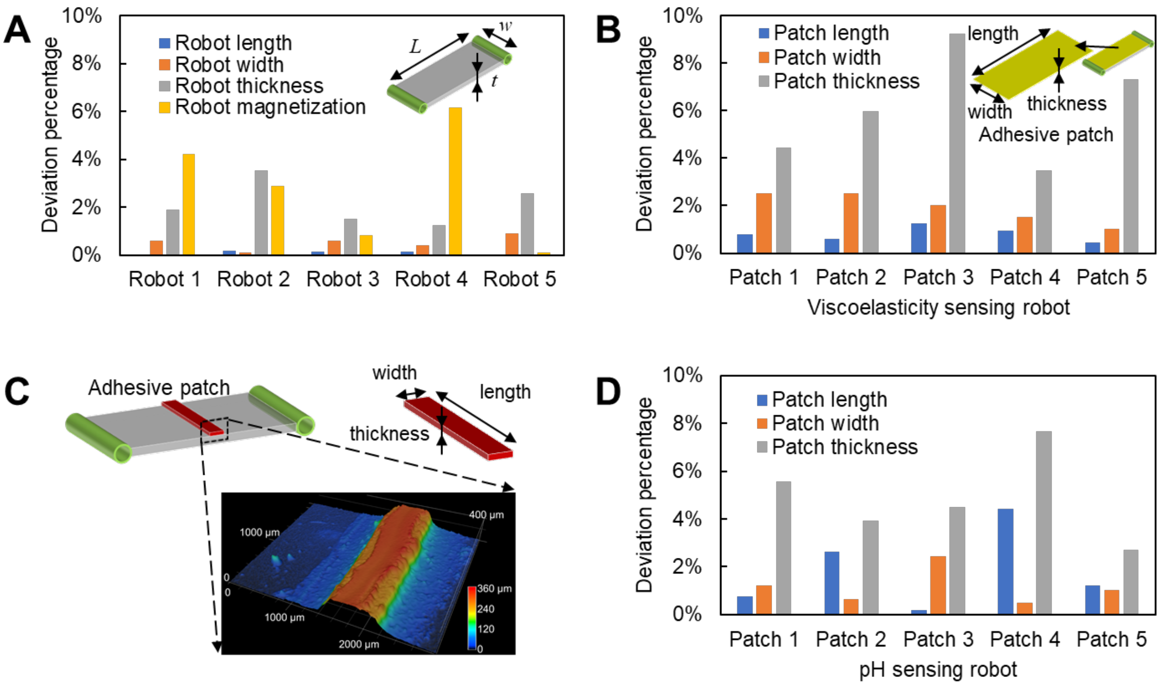

Fig. S16. Quantitative characterization of the robot fabrication. The deviation percentage values were calculated with respect to the average value of the measured values. **A.** The deviation in the dimensions and the magnetization of 5 fabricated robots. The length and width of the robots were measured using a caliper and the thickness of the robots was measured using a 3D laser scanning confocal microscope (VK-X260K, KEYENCE). The remanent magnetization was measured using the vibrating sample magnetometer (EZ7 VSM, MicroSense LLC). **B.** The deviation in the dimensions of the adhesive patches of 5 fabricated viscoelasticity sensing robots. The length and width were measured using a caliper and the thickness was measured using a 3D laser scanning confocal microscope. **C.** The schematic of the adhesive patch of a pH sensing robot with the surface profile as measured using a 3D laser scanning confocal microscope. **D.** The deviation in the dimensions of the adhesive patches of 5 fabricated pH sensing robots.

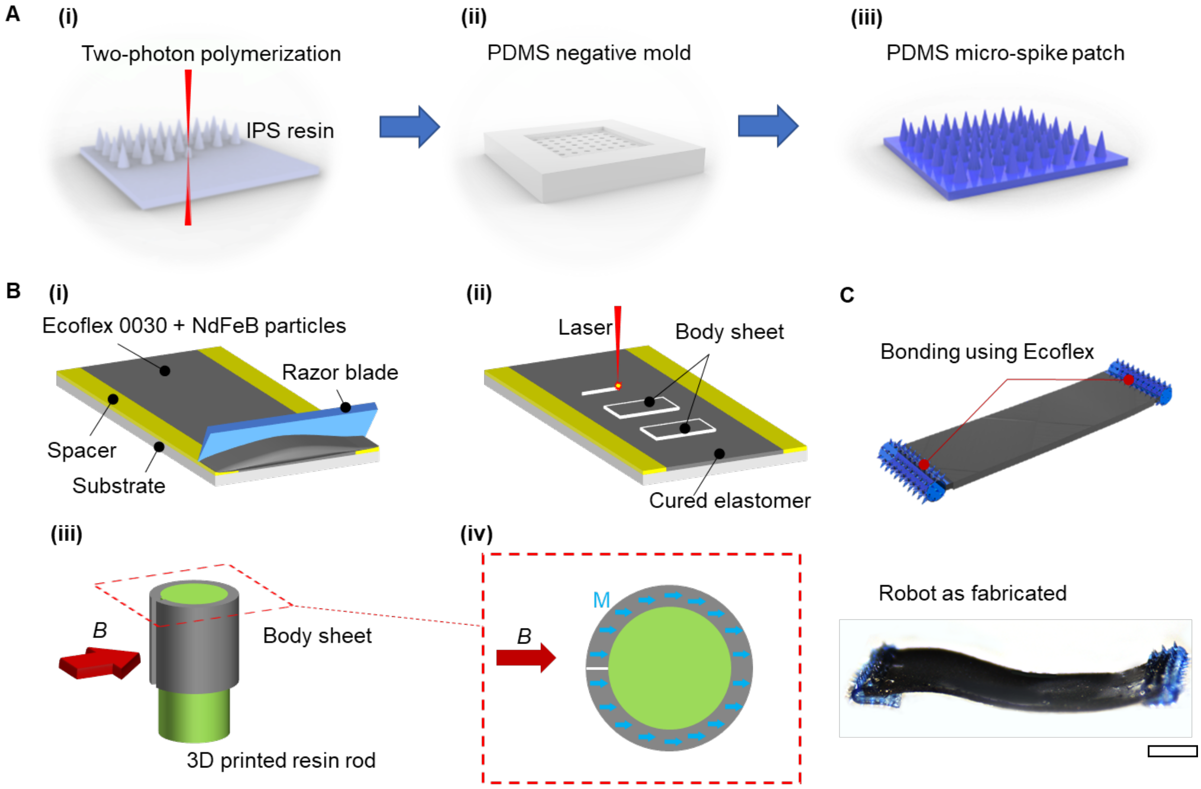

Fig. S17. Schematics of the fabrication process of the robot. **A**. Illustration of the fabrication of the robot pad. **(i)**. Schematic of 3D printing the master mold using the Two-Photon-Polymerization (2PP). The master mold of the micro-spikes was prepared using a 2PP 3D micro-printer (Photonic Professional GT, Nanoscribe GmbH) with a rigid IP-S commercial photoresist (Nanoscribe GmbH). **(ii)**. Illustration of the PDMS negative mold for the micro-spike pad. The mold was obtained by molding 10:1 PDMS solution against the post-processed 3D printed IP-S master molds. **(iii).** Illustration of the 20:1 PDMS micro-spike pad patch. This patch was fabricated by molding 20:1 PDMS solution against the post-processed PDMS negative molds. **B.** Illustration of fabricating a ferromagnetic-elastic sheet with a desired thickness. **(i)**. The mixture of NdFeB microparticles and Ecoflex-0030 elastomer was poured onto a PMMA substrate, after which a razor blade scratched against the spacer for a uniform sheet thickness. **(ii)**. Illustration of the fabrication of the robot body. The ferromagnetic-elastic sheet was cut into the rectangular segment with specified dimensions using a laser machine (LPKF ProtoLaser U3, LPKF Laser & Electronics AG). **(iii)**. Schematic of the magnetization process for the ferromagnetic-elastic sheet of the robot body. The trimmed rectangular sheet was wrapped over a 3D printed cylindrical rod of 2.1 mm diameter and 5 mm length and then placed into a uniform magnetizing filed of 1.8 T (EZ7 VSM, MicroSense LLC) with the two ends facing to the north pole. **(iv)**. Illustration of the magnetized robot body. The blue arrows indicate the magnetization profile. **C.** Illustration of the assembly process of the robot pads and the robot body and the corresponding soft robot as fabricated. The body and pads were bonded using Ecoflex-0030, and the robot pads were coated by bioadhesives. Scale bar, 1 mm.

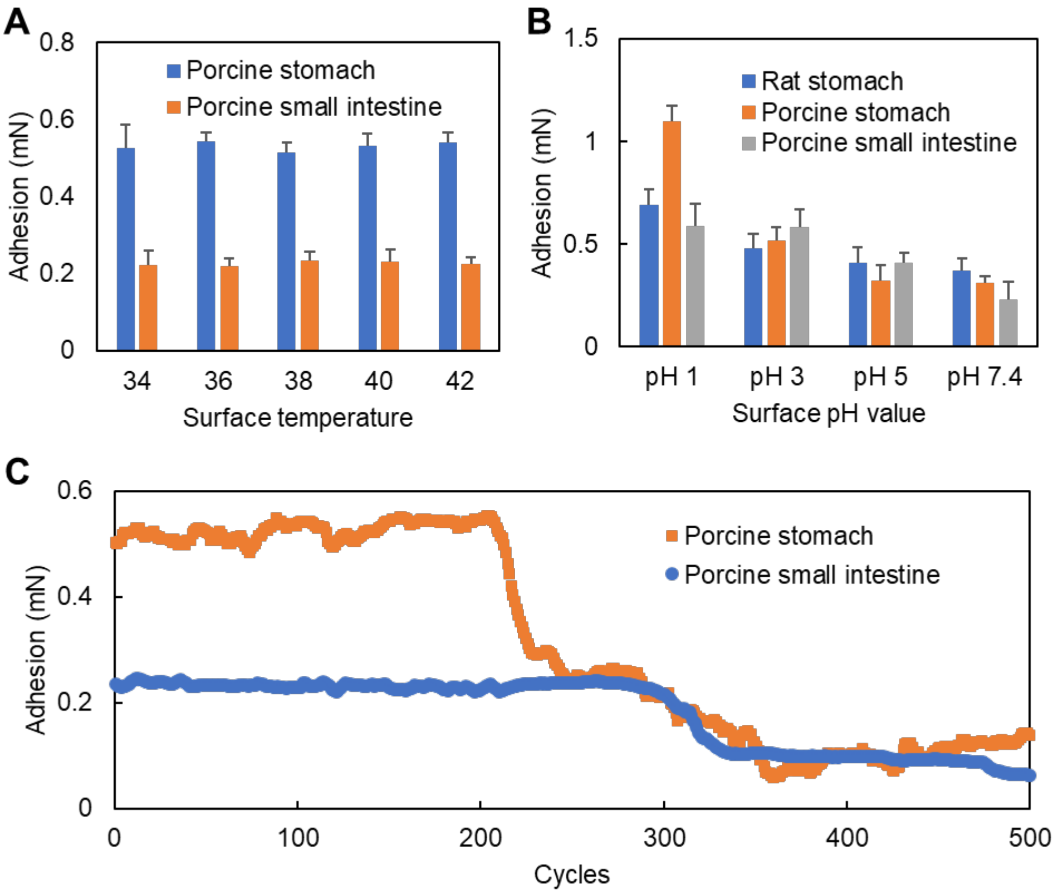

Fig. S18. The characterization of the pH-responsive bioadhesives. A. The bioadhesive-tissue adhesion on different tissue surfaces under various temperature conditions. **B.** The bioadhesive-tissue adhesion on various tissue surfaces of specific pH values. The standard phosphate-buffered saline (PBS) buffer solution of different pH values was added onto the same tissue surfaces to achieve the prescribed pH value. **C.** Adhesion force of the bioadhesives on various porcine tissues as a function of the loading-peeling cycle number. The measurement conditions include preload: 0.1 mN, contact time: 20 second. The error bar in **A** and **B** represents the standard deviation for n = 5 measurements, where n represents the number of samples.

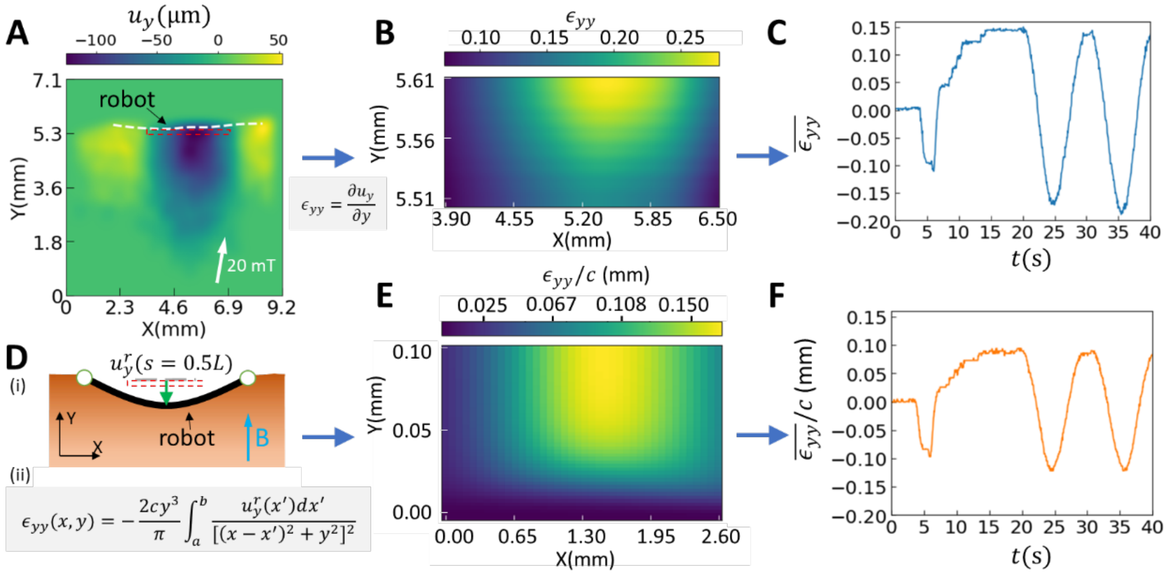

Fig. S19. Determination of the correlation coefficient c between the robot displacement and the strain field of the substrate material for viscoelasticity sensing. A. The material displacement u_y field, characterized by the digital imaging correlation analysis (see **Section 10 of Material and Methods).** The white dashed line indicates the initial robot curve without the magnetic actuation. The data within the region denoted by the red dashed rectangle (2.6 mm × 0.1 mm) was adopted for calibrating c. **B.** The strain field of the substrate material calculated from the u_y field within the red rectangle with $\epsilon_{yy} = \partial u_y / \partial y$. **C.** The mean strain over the region in **B**, $\overline{\epsilon_{yy}}$ as a function of time. **D.** The schematics of computing the material ϵ_{yy} from the robot displacement u_y^r. **(i)** The schematics of the robot displacement u_y^r. The green arrow indicates $u_y^r(s = 0.5L)$, where L is the robot length (6.5 mm). The data within the region denoted by the red dashed rectangle (2.6 mm × 0.1 mm) was adopted for calibrating c by regression. **(ii)** The equation for computing ϵ_{yy} from u_y^r ("**Estimating tissue viscoelastic properties**" Section in **Materials and Methods) E.** The strain field $\epsilon_{yy,r}/c$ of the substrate material computed from the robot curve displacement. **F.** The mean value $\overline{\epsilon_{yy}}/c$ over the region in **E** as a function of time. c can be calibrated by regression between $\overline{\epsilon_{yy}}$ and $\overline{\epsilon_{yy}}/c$ on different materials as 1.503 mm^{-1} with a standard deviation of 0.188 mm^{-1}. The material used for calibration includes 0.4% agarose gel, 0.5% agarose gel and 60-0.3 agarose-based gel (weight ratio, sucrose: water: agarose = 60: 40: 0.3), and 10 samples were prepared for calibration.

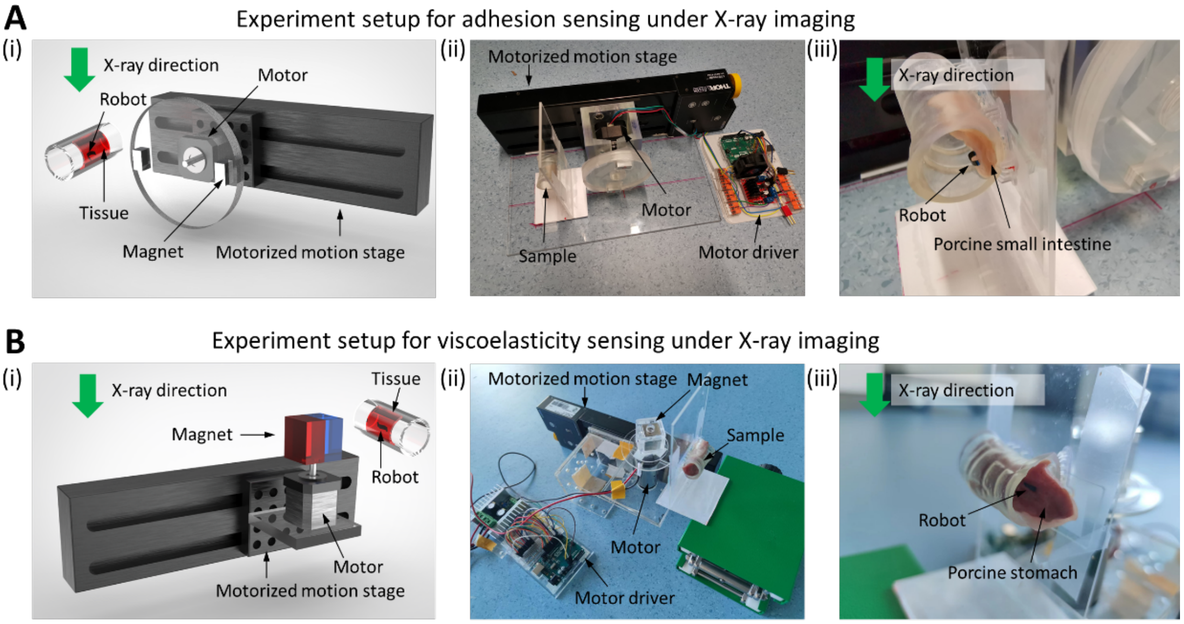

Fig. S20. The experimental setups for sensing adhesion and viscoelasticity inside an X-ray cabinet. **A.** The schematic **(i)** and the practical image **(ii)** of the experimental setup for adhesion sensing under X-ray imaging. The robot and porcine small intestine tissue were placed inside a 3D printed human intestine phantom (Elastic 50A, Formlabs Inc., density 1 g/cm^3, the same as the tissues) as illustrated in **(iii)**. Two magnets (Product number: 3800, EarthMag GmbH, Dortmund, Germany) were fixed at the two ends of a PMMA disk of 10 cm in diameter with the opposite magnetic field directions. The disk was connected to a step motor (NEMA17-01, Neukirchen-Vluyn, Germany) which was mounted on a linear motorized stage (LTS300/M, Thorlabs Inc., Newton, NJ, USA). **B.** The schematic **(i)** and the practical image **(ii)** of the experimental setup for viscoelasticity sensing under X-ray imaging. The robot and porcine stomach tissue were placed inside a 3D printed human intestine phantom (Elastic 50A, Formlabs Inc., density 1 g/cm^3, the same as the tissues) as illustrated in **(iii)**. A magnet (Product number: 3982, EarthMag GmbH, Dortmund, Germany) was connected to a DC motor (242478, Maxon Co., Switzerland) which was mounted on a linear motorized stage (LTS300/M, Thorlabs Inc., Newton, NJ, USA) as shown in **(i)**.

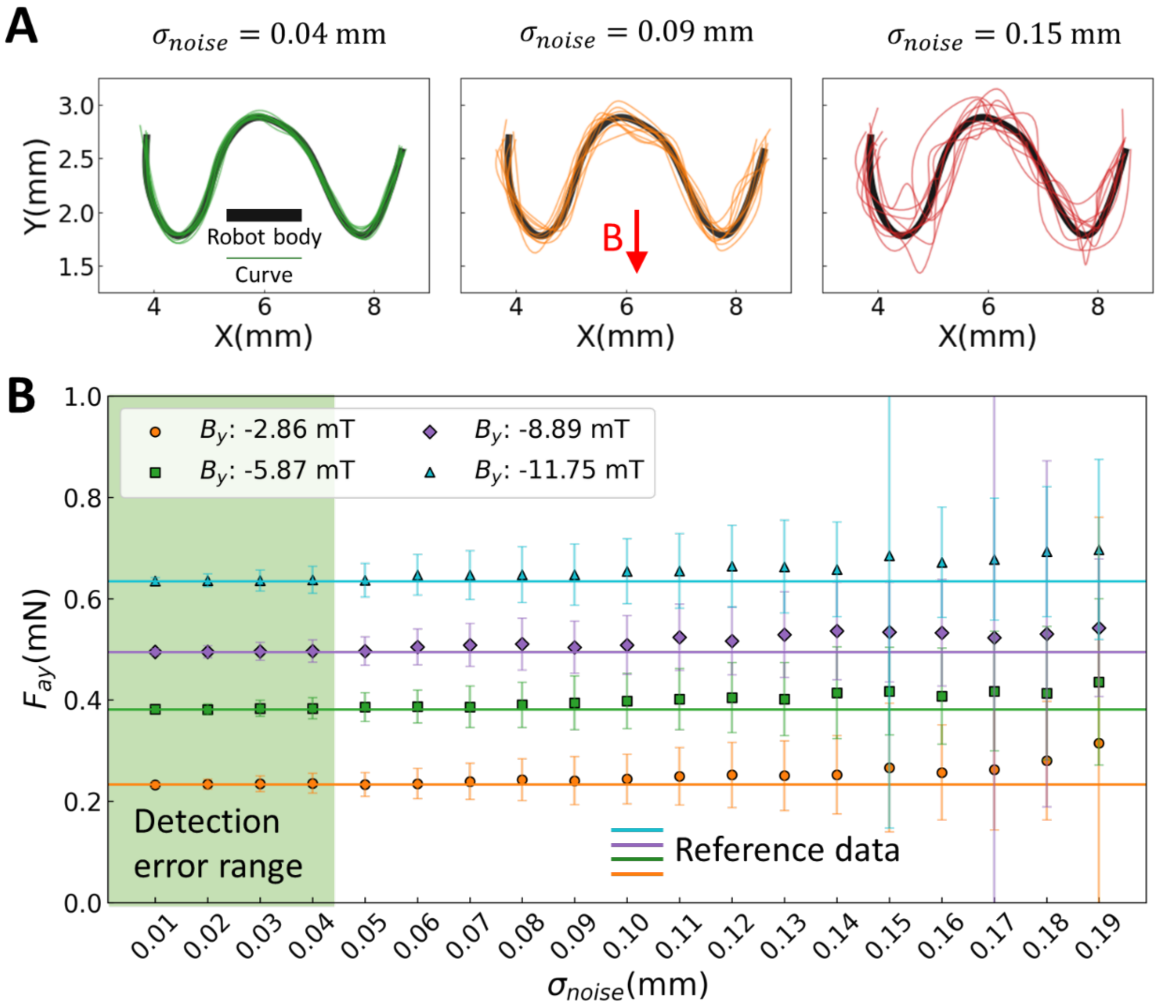

Fig. S21. The effect of the detection noise on the adhesion estimation. A. The superposition of the robot curve and the noisy curve of various levels. Random noise subject to the normal distribution with the standard deviation σ_{noise} is added to the anchor points of the robot centerline extracted by the image processing, which is further used to fit the B-spline as the robot curve. The width of the black curve is set the same as the practical robot body thickness of 0.15 mm. **B.** The correlation between the estimated adhesion and σ_{noise}, where the detection error is within the σ_{noise} of 0.045 mm. Error bars represent the standard deviation of the estimated F_{ay}.

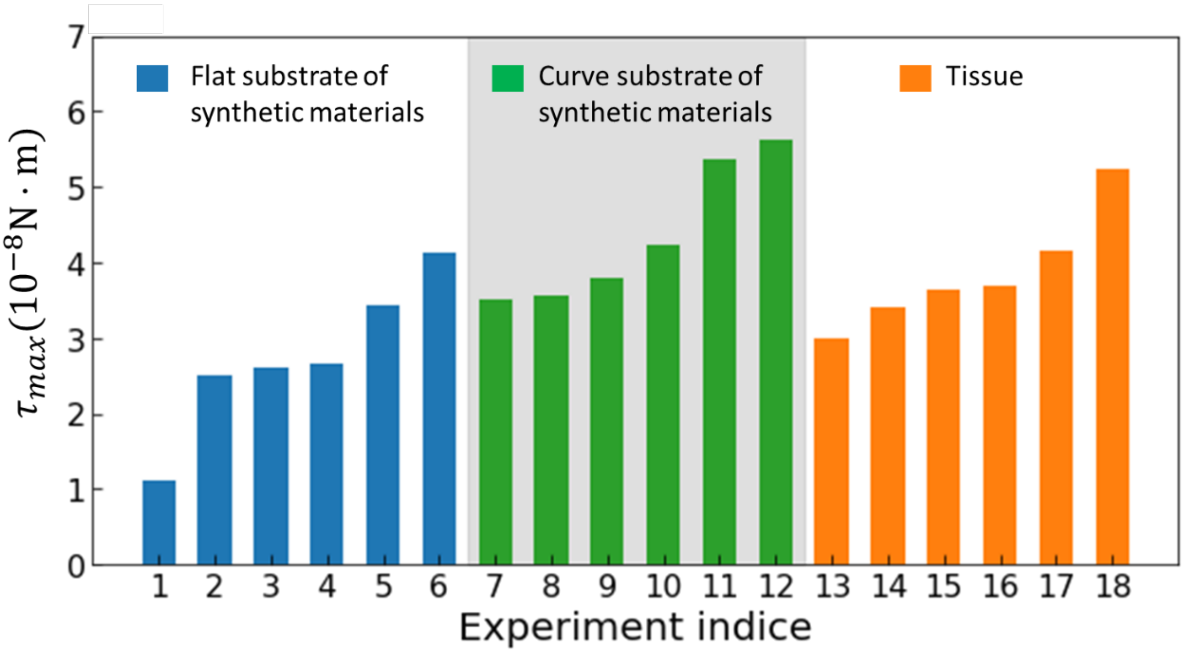

Fig. S22. The estimated maximum reaction torque at the adhesive patch and the substrate material interface during adhesion sensing. The maximum six estimated torques for three experimental groups, including the flat substrate of synthetic materials, curve substrate of synthetic materials, and tissue. The torque is negligible compared with the magnitude of the adhesion.

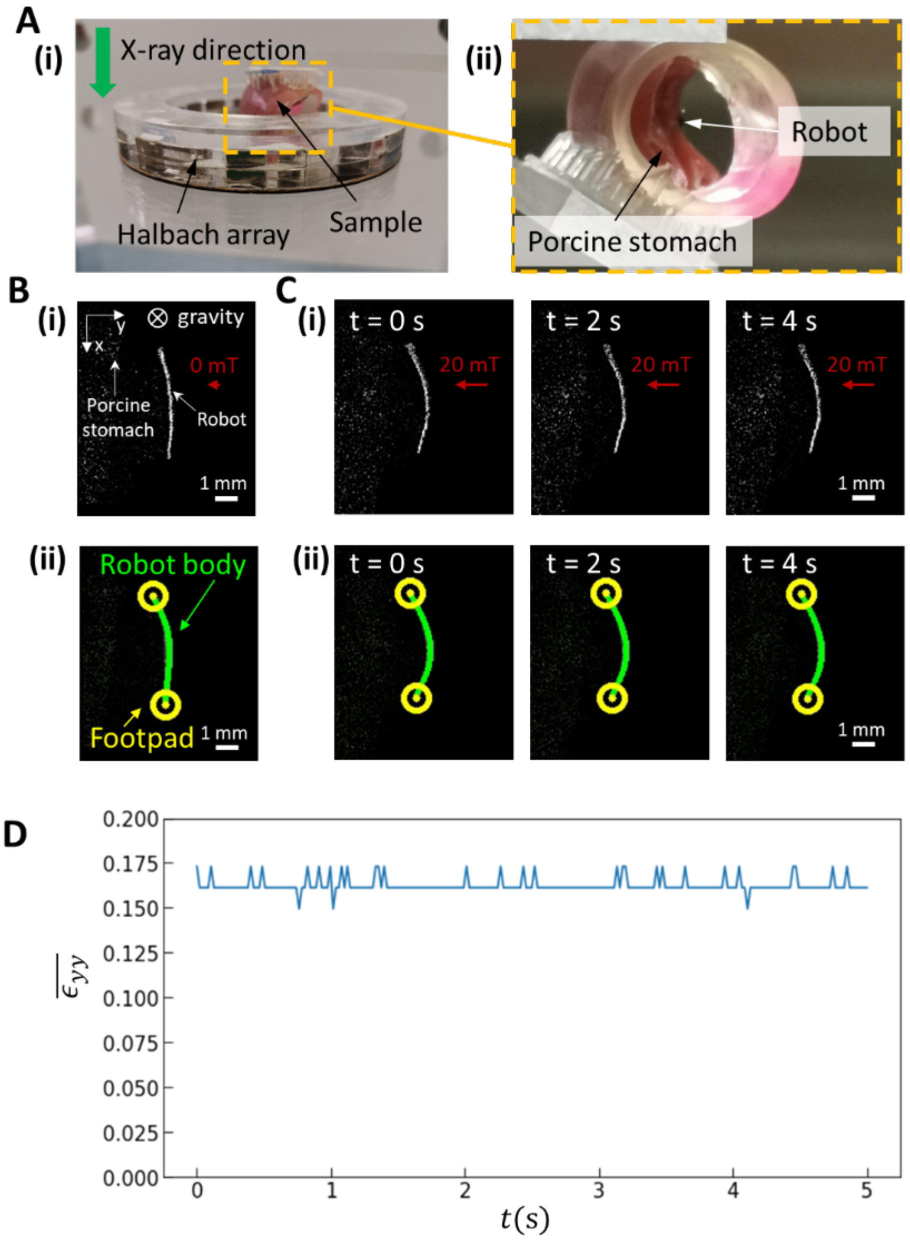

Fig. S23. Calculating the signal-to-noise ratio. A. The experimental setup **(i)** for calculating the signal-to-noise ratio. The robot and porcine stomach tissue were placed inside a 3D printed human gastrointestinal phantom (Elastic 50A, Formlabs Inc., density 1 g/cm^3, the same as the tissues), as illustrated in **(ii)**. **B.** The robot body **(i)** under X-ray imaging (XPERT 80, KUBTEC, Stratford CT) with a voltage of 68 kV, a current of 100 μA and a speed of 30 frames per second. The extracted robot body shape **(ii)** with the image processing algorithm in "**Robot shape tracking and analysis**" Section in **Materials and Methods**. **C.** The robot body **(i)** visualized by X-ray imaging under the actuation of a static magnetic field (20 mT). The small variations of the extracted robot body shape **(ii)** due to the imaging noise. **D.** The estimated tissue strain based on the extracted robot body shape under the actuation of a static magnetic field. $\overline{\epsilon_{yy}} = \int_{0.3L}^{0.7L} \epsilon_{yy}(s)ds\,/0.4L$, where L is the robot length (6.5 mm). In all figures, scale bars are 1 mm.

Supplementary Movies

Movie S1. Robot locomotion and deployment on tissues.

This video shows the process of deploying the robot to the tissue with the climbing locomotion, sensing the tissue adhesion and viscoelasticity with static and dynamic shape change, and retrieving the robot from the tissue surfaces ex vivo. The robot locomotion and sensing function are controlled by varying the external magnetic field.

Movie S2. Robot sensing adhesion on synthetic materials and tissues.

This video demonstrates the mechanism of sensing adhesion using the robot static body shape, the test results on synthetic materials of different types and curvatures, and the ability to sense pH on tissue surfaces by integrating the pH-responsive adhesive patch.

Movie S3. Robot sensing viscoelasticity on synthetic materials and tissues.

This video presents the mechanism for sensing material viscoelasticity with the robot dynamic body shape and the test results on different synthetic materials and ex vivo tissues. The generated displacement and strain field by tracking the fluorescent particles dispersed in the material are also shown to visualize the material deformation. Additionally, the video also shows a proof-of-concept demonstration of sensing stiffer artificial disease spots using the robot dynamic body shape.

Movie S4. Robot sensing pH of TNK1-expression disease models of mice ex vivo.

This video shows the X-ray imaging quality for sensing tissue adhesion ex vivo. This video also shows the results of sensing adhesion on mice stomach tissues with and without a TNK1-expression disease ex vivo.

Movie S5. Robot sensing viscoelasticity of TNK1-expression disease models of mice ex vivo.

This video illustrates the X-ray and ultrasound imaging effect for sensing tissue viscoelasticity and the viscoelasticity sensing results on ex vivo mice stomach tissues with and without a TNK1-expression disease.

REFERENCES AND NOTES

1. A. J. Thompson, E. K. Pillai, I. B. Dimov, S. K. Foster, C. E. Holt, K. Franze, Rapid changes in tissue mechanics regulate cell behaviour in the developing embryonic brain. *eLife* **8**, e39356 (2019).

2. A. Dance, The secret forces that squeeze and pull life into shape. *Nature* **589**, 186–188 (2021).

3. N. F. Läubli, J. T. Burri, J. Marquard, H. Vogler, G. Mosca, N. Vertti-Quintero, N. Shamsudhin, A. deMello, U. Grossniklaus, D. Ahmed, B. J. Nelson, 3D mechanical characterization of single cells and small organisms using acoustic manipulation and force microscopy. *Nat. Commun.* **12**, 2583 (2021).

4. M. Lin, H. Hu, S. Zhou, S. Xu, Soft wearable devices for deep-tissue sensing. *Nat. Rev. Mater.* **7**, 850–869 (2022).

5. E. Song, Y. Huang, N. Huang, Y. Mei, X. Yu, J. A. Rogers, Recent advances in microsystem approaches for mechanical characterization of soft biological tissues. *Microsyst. Nanoeng.* **8**, 77 (2022).

6. K. M. Kennedy, L. Chin, R. A. McLaughlin, B. Latham, C. M. Saunders, D. D. Sampson, B. F. Kennedy, Quantitative micro-elastography: Imaging of tissue elasticity using compression optical coherence elastography. *Sci. Rep.* **5**, 15538 (2015).

7. R. M. S. Sigrist, J. Liau, A. El Kaffas, M. C. Chammas, J. K. Willmann, Ultrasound elastography: Review of techniques and clinical applications. *Theranostics* **7**, 1303–1329 (2017).

8. K. K. Shung, *Diagnostic Ultrasound: Imaging and Blood Flow Measurements* (CRC Press, 2005).

9. G. Low, S. A. Kruse, D. J. Lomas, General review of magnetic resonance elastography. *World J. Radiol.* **8**, 59–72 (2016).

10. F. Schrank, C. Warmuth, S. Görner, T. Meyer, H. Tzschätzsch, J. Guo, Y. O. Uca, T. Elgeti, J. Braun, I. Sack, Real-time MR elastography for viscoelasticity quantification in skeletal muscle during dynamic exercises. *Magn. Reson. Med.* **84**, 103–114 (2020).

11. H. Hu, X. Zhu, C. Wang, L. Zhang, X. Li, S. Lee, Z. Huang, R. Chen, Z. Chen, C. Wang, Y. Gu, Y. Chen, Y. Lei, T. Zhang, N. H. Kim, Y. Guo, Y. Teng, W. Zhou, Y. Li, A. Nomoto, S. Sternini, Q. Zhou, M. Pharr, F. L. di Scalea, S. Xu, Stretchable ultrasonic transducer arrays for three-dimensional imaging on complex surfaces. *Sci. Adv.* **4**, eaar3979 (2018).

12. C. Wang, X. Li, H. Hu, L. Zhang, Z. Huang, M. Lin, Z. Zhang, Z. Yin, B. Huang, H. Gong, S. Bhaskaran, Y. Gu, M. Makihata, Y. Guo, Y. Lei, Y. Chen, C. Wang, Y. Li, T. Zhang, Z. Chen, A. P. Pisano, L. Zhang, Q. Zhou, S. Xu, Monitoring of the central blood pressure waveform via a conformal ultrasonic device. *Nat. Biomed. Eng.* **2**, 687–695 (2018).

13. C. Wang, X. Chen, L. Wang, M. Makihata, H.-C. Liu, T. Zhou, X. Zhao, Bioadhesive ultrasound for long-term continuous imaging of diverse organs. *Science* **377**, 517–523 (2022).

14. X. Yu, H. Wang, X. Ning, R. Sun, H. Albadawi, M. Salomao, A. C. Silva, Y. Yu, L. Tian, A. Koh, C. M. Lee, A. Chempakasseril, P. Tian, M. Pharr, J. Yuan, Y. Huang, R. Oklu, J. A. Rogers, Needle-shaped ultrathin piezoelectric microsystem for guided tissue targeting via mechanical sensing. *Nat. Biomed. Eng.* **2**, 165–172 (2018).

15. E. Song, Z. Xie, W. Bai, H. Luan, B. Ji, X. Ning, Y. Xia, J. M. Baek, Y. Lee, R. Avila, H.-Y. Chen, J.-H. Kim, S. Madhvapathy, K. Yao, D. Li, J. Zhou, M. Han, S. M. Won, X. Zhang, D. J. Myers, Y. Mei, X. Guo, S. Xu, J.-K. Chang, X. Yu, Y. Huang, J. A. Rogers, Miniaturized electromechanical devices for the characterization of the biomechanics of deep tissue. *Nat. Biomed. Eng.* **5**, 759–771 (2021).

16. J. Shin, Y. Yan, W. Bai, Y. Xue, P. Gamble, L. Tian, I. Kandela, C. R. Haney, W. Spees, Y. Lee, M. Choi, J. Ko, H. Ryu, J. K. Chang, M. Pezhouh, S. K. Kang, S. M. Won, K. J. Yu, J. Zhao, Y. K. Lee, M. MacEwan, S. K. Song, Y. Huang, W. Z. Ray, J. A. Rogers, Bioresorbable pressure sensors protected with thermally grown silicon dioxide for the monitoring of chronic diseases and healing processes. *Nat. Biomed. Eng.* **3**, 37–46 (2019).

17. A. J. Bredenoord, Impedance-pH monitoring: New standard for measuring gastro-oesophageal reflux. *Neurogastroenterol. Motil.* **20**, 434–439 (2008).

18. G. Ciuti, R. Caliò, D. Camboni, L. Neri, F. Bianchi, A. Arezzo, A. Koulaouzidis, S. Schostek, D. Stoyanov, C. M. Oddo, B. Magnani, A. Menciassi, M. Morino, M. O. Schurr, P. Dario, Frontiers of robotic endoscopic capsules: A review. *J. Microbio Robot.* **11**, 1–18 (2016).

19. J. Min, Y. Yang, Z. Wu, W. Gao, Robotics in the gut. *Adv. Ther.* **3**, 1900125 (2020).

20. N. Shamsudhin, V. I. Zverev, H. Keller, S. Pane, P. W. Egolf, B. J. Nelson, A. M. Tishin, Magnetically guided capsule endoscopy. *Med. Phys.* **44**, e91–e111 (2017).

21. S. Baltsavias, W. Van Treuren, M. J. Weber, J. Charthad, S. Baker, J. L. Sonnenburg, A. Arbabian, In vivo wireless sensors for gut microbiome redox monitoring. *IEEE Trans. Biomed. Eng.* **67**, 1821–1830 (2020).

22. F. Serwane, A. Mongera, P. Rowghanian, D. A. Kealhofer, A. A. Lucio, Z. M. Hockenbery, O. Campàs, In vivo quantification of spatially varying mechanical properties in developing tissues. *Nat. Methods* **14**, 181–186 (2016).

23. F. E. Uslu, C. D. Davidson, E. Mailand, N. Bouklas, B. M. Baker, M. S. Sakar, Engineered extracellular matrices with integrated wireless microactuators to study mechanobiology. *Adv. Mater.* **33**, 2102641 (2021).

24. Y. Wu, X. Dong, J. K. Kim, C. Wang, M. Sitti, Wireless soft millirobots for climbing three-dimensional surfaces in confined spaces. *Sci. Adv.* **8**, eabn3431 (2022).

25. N. D. Blelloch, H. J. Yarbrough, K. A. Mirica, Stimuli-responsive temporary adhesives: Enabling debonding on demand through strategic molecular design. *Chem. Sci.* **12**, 15183–15205 (2021).

26. D. Yan, M. Pezzulla, L. Cruveiller, A. Abbasi, P. M. Reis, Magneto-active elastic shells with tunable buckling strength. *Nat. Commun.* **12**, 2831 (2021).

27. J. Li, A. D. Celiz, J. Yang, Q. Yang, I. Wamala, W. Whyte, B. R. Seo, N. V. Vasilyev, J. J. Vlassak, Z. Suo, D. J. Mooney, Tough adhesives for diverse wet surfaces. *Science* **357**, 378–381 (2017).

28. J. Huang, Y. Liu, Y. Yang, Z. Zhou, J. Mao, T. Wu, J. Liu, Q. Cai, C. Peng, Y. Xu, B. Zeng, W. Luo, G. Chen, C. Yuan, L. Dai, Electrically programmable adhesive hydrogels for climbing robots. *Sci. Robot.* **6**, eabe1858 (2021).

29. M. S. Akram Bhuiyan, J. D. Roland, B. Liu, M. Reaume, Z. Zhang, J. D. Kelley, B. P. Lee, In situ deactivation of catechol-containing adhesive using electrochemistry. *J. Am. Chem. Soc.* **142**, 4631–4638 (2020).

30. T. Machida, A study of intragastric pH in patients with peptic ulcer-with special reference to the clinical significance of basal pH value. *Gastroenterol. Jpn.* **16**, 447–458 (1981).

31. A. M. Handorf, Y. Zhou, M. A. Halanski, W.-J. Li, Tissue stiffness dictates development, homeostasis, and disease progression. *Organogenesis* **11**, 1–15 (2015).

32. D. A. Stoltz, D. K. Meyerholz, M. J. Welsh, Origins of cystic fibrosis lung disease. *N. Engl. J. Med.* **372**, 351–362 (2015).

33. N. Miyahara, T. Kokubo, Y. Hara, A. Yamada, T. Koike, Y. Arai, Evaluation of X-ray doses and their corresponding biological effects on experimental animals in cone-beam micro-CT scans (R-mCT2). *Radiol. Phys. Technol.* **9**, 60–68 (2016).

34. M. Armacki, A. K. Trugenberger, A. K. Ellwanger, T. Eiseler, C. Schwerdt, L. Bettac, D. Langgartner, N. Azoitei, R. Halbgebauer, R. Groß, T. Barth, A. Lechel, B. M. Walter, J. M. Kraus, C. Wiegreffe, J. Grimm, A. Scheffold, M. R. Schneider, K. Peuker, S. Zeißig, S. Britsch, S. Rose-John, S. Vettorazzi, E. Wolf, A. Tannapfel, K. Steinestel, S. O. Reber, P. Walther, H. A. Kestler, P. Radermacher, T. F. E. Barth, M. Huber-Lang, A. Kleger, T. Seufferlein, Thirty-eight-negative kinase 1 mediates trauma-induced intestinal injury and multi-organ failure. *J. Clin. Investig.* **128**, 5056–5072 (2018).

35. J. Zhang, Y. Guo, W. Hu, R. H. Soon, Z. S. Davidson, M. Sitti, Liquid crystal elastomer-based magnetic composite films for reconfigurable shape-morphing soft miniature machines. *Adv. Mater.* **33**, 2006191 (2021).

36. A. Thitaikumar, T. A. Krouskop, J. Ophir, Signal-to-noise ratio, contrast-to-noise ratio and their trade-offs with resolution in axial-shear strain elastography. *Phys. Med. Biol.* **52**, 13–28 (2007).

37. R. Delaunay, Y. Hu, T. Vercauteren, in *Medical Image Computing and Computer Assisted Intervention–MICCAI 2020* (Springer, 2020), pp. 573–582.

38. M. Mirzaei, A. Asif, H. Rivaz, Combining total variation regularization with window-based time delay estimation in ultrasound elastography. *IEEE Trans. Med. Imaging* **38**, 2744–2754 (2019).

39. S.-J. Kim, D.-S. Lee, I.-G. Kim, D.-W. Sohn, J.-Y. Park, B.-K. Choi, S.-W. Kim, Evaluation of the biocompatibility of a coating material for an implantable bladder volume sensor. *Kaohsiung J. Med. Sci.* **28**, 123–129 (2012).

40. H.-J. Chung, A. M. Parsons, L. Zheng, Magnetically controlled soft robotics utilizing elastomers and gels in actuation: A review. *Adv. Intell. Syst.* **3**, 2000186 (2021).

41. D. Armstrong, Review article: Gastric pH—The most relevant predictor of benefit in reflux disease? *Aliment. Pharmacol. Ther.* **20**, 19–26 (2004).

42. T. Ebihara, N. Venkatesan, R. Tananka, M. S. Ludwig Changes in extracellular matrix and tissue viscoelasticity in bleomycin-induced lung fibrosis. *Am. J. Respir. Crit. Care Med.* **162**, 1569–1576 (2000).

43. H. T. Nia, L. L. Munn, R. K. Jain, Physical traits of cancer. *Science* **370**, eaaz0868 (2020).

44. Merki, H. S., C. J. Fimmel, R. P. Walt, K. Harre, J. Röhmel, L. Witzel, Pattern of 24 hour intragastric acidity in active duodenal ulcer disease and in healthy controls. *Gut* **29**, 1583–1587 (1988).

45. A. R. Narkar, C. Kendrick, K. Bellur, T. Leftwich, Z. Zhang, B. P. Lee, Rapidly responsive smart adhesive-coated micropillars utilizing catechol–boronate complexation chemistry. *Soft Matter* **15**, 5474–5482 (2019).

46. S. Maurer, A. Junghans, T. A. Vilgis, Impact of xanthan gum, sucrose and fructose on the viscoelastic properties of agarose hydrogels. *Food Hydrocoll.* **29**, 298–307 (2012).

47. P. Virtanen, R. Gommers, T. E. Oliphant, M. Haberland, T. Reddy, D. Cournapeau, E. Burovski, P. Peterson, W. Weckesser, J. Bright, S. J. van der Walt, M. Brett, J. Wilson, K. J. Millman, N. Mayorov, A. R. J. Nelson, E. Jones, R. Kern, E. Larson, C. J. Carey, I. Polat, Y. Feng, E. W. Moore, J. VanderPlas, D. Laxalde, J. Perktold, R. Cimrman, I. Henriksen, E A Quintero, C. R. Harris, A. M. Archibald, A. H. Ribeiro, F. Pedregosa, P. van Mulbregt; SciPy 1.0 Contributors, SciPy 1.0: Fundamental algorithms for scientific computing in Python. *Nat. Methods* **17**, 261–272 (2020).

48. J. Nocedal, S. J. Wright, *Numerical Optimization* (Springer, 1999).

49. Y. Kim, H. Yuk, R. Zhao, S. A. Chester, X. Zhao, Printing ferromagnetic domains for untethered fast-transforming soft materials. *Nature* **558**, 274–279 (2018).

50. K. L. Johnson, *Contact Mechanics* (Cambridge Univ. Press, 1987).
